# Supplementary material for: Lgr5 and Col22a1 Mark Progenitor Cells in the Lineage toward Juvenile Articular Chondrocytes
Source: Stem Cell Reports. 2019 Sep 12;13(4):713–29. doi: 10.1016/j.stemcr.2019.08.006 (PMC6829785; doi:10.1016/j.stemcr.2019.08.006)
Supplement: Document S1. Figures S1–S6 and Tables S1–S7 [file mmc1.pdf]

Stem Cell Reports, Volume *13*

Supplemental Information

Lgr5 and *Col22a1* Mark Progenitor Cells in the Lineage toward Juvenile Articular Chondrocytes

Chen Feng, Wilson Cheuk Wing Chan, Yan Lam, Xue Wang, Peikai Chen, Ben Niu, Vivian Chor Wing Ng, Jia Chi Yeo, Sigmar Stricker, Kathryn Song Eng Cheah, Manuel Koch, Stefan Mundlos, Huck Hui Ng, and Danny Chan

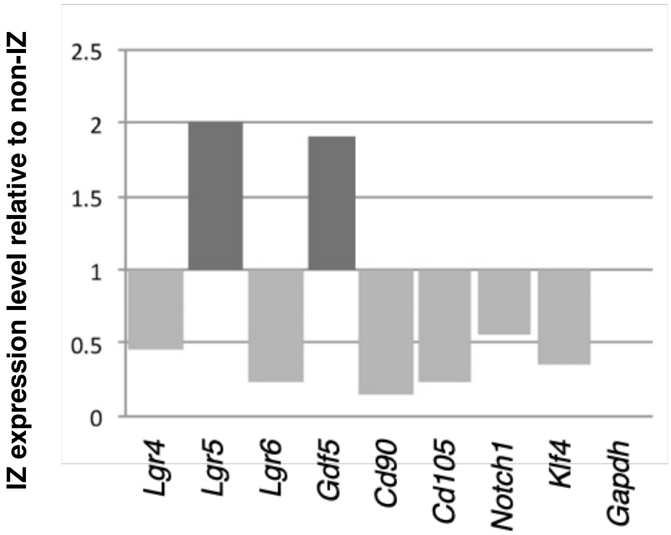

Figure S1. Quantitative PCR showed expression of Gdf5 and Lgr5 in digit interzone mRNA was extracted from the digit interzone (IZ) and surrounding non-interzone regions of a E14.5 embryo to generate cDNA for QPCR. Expression of *Gapdh* was normalized to 1.0, values higher than 1.0 indicates these genes are upregulated, vice versa. Among all the selected stem cell/progenitor markers, known marker of joint interzone *Gdf5* and novel marker identified in this paper *Lgr5* were upregulated in the interzone region.

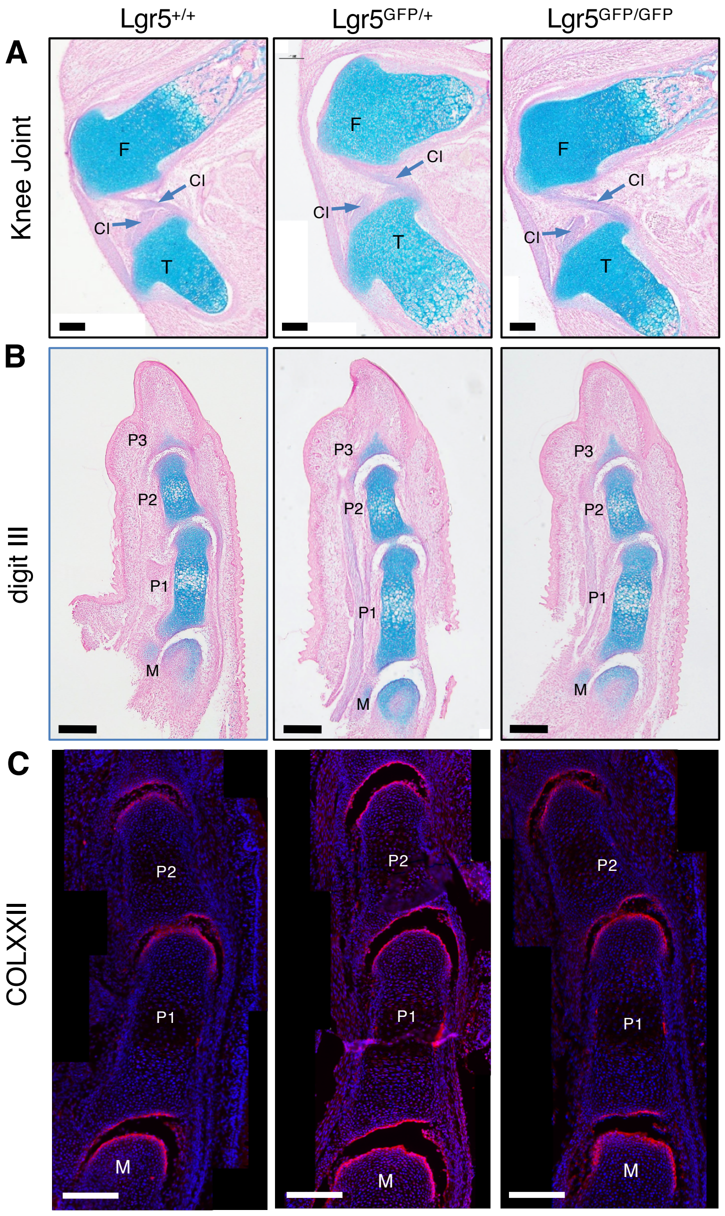

Figure S2. ***Lgr5-GFP*** **mutant developed normal knee and digit joints**
Representative histology sections stained with Alcian blue and nuclear fast red, showing no observable difference between the developing knee **(A)** and digit joints **(B)** of Lgr5$^{+/+}$, Lgr5$^{GFP/+}$ and Lgr5$^{GFP/GFP}$ E18.5 embryos (n=3 per genotype). **(C)** The articular cartilages appeared to have formed normally with the expected expression and localization of COLXXII in the superficial layer. F=femur, T=tibia, Cl=cruciate ligament, M=metacarpal, P1= proximal phalange, P2=middle phalange, P3=distal phalange. Scale bar, 200μm

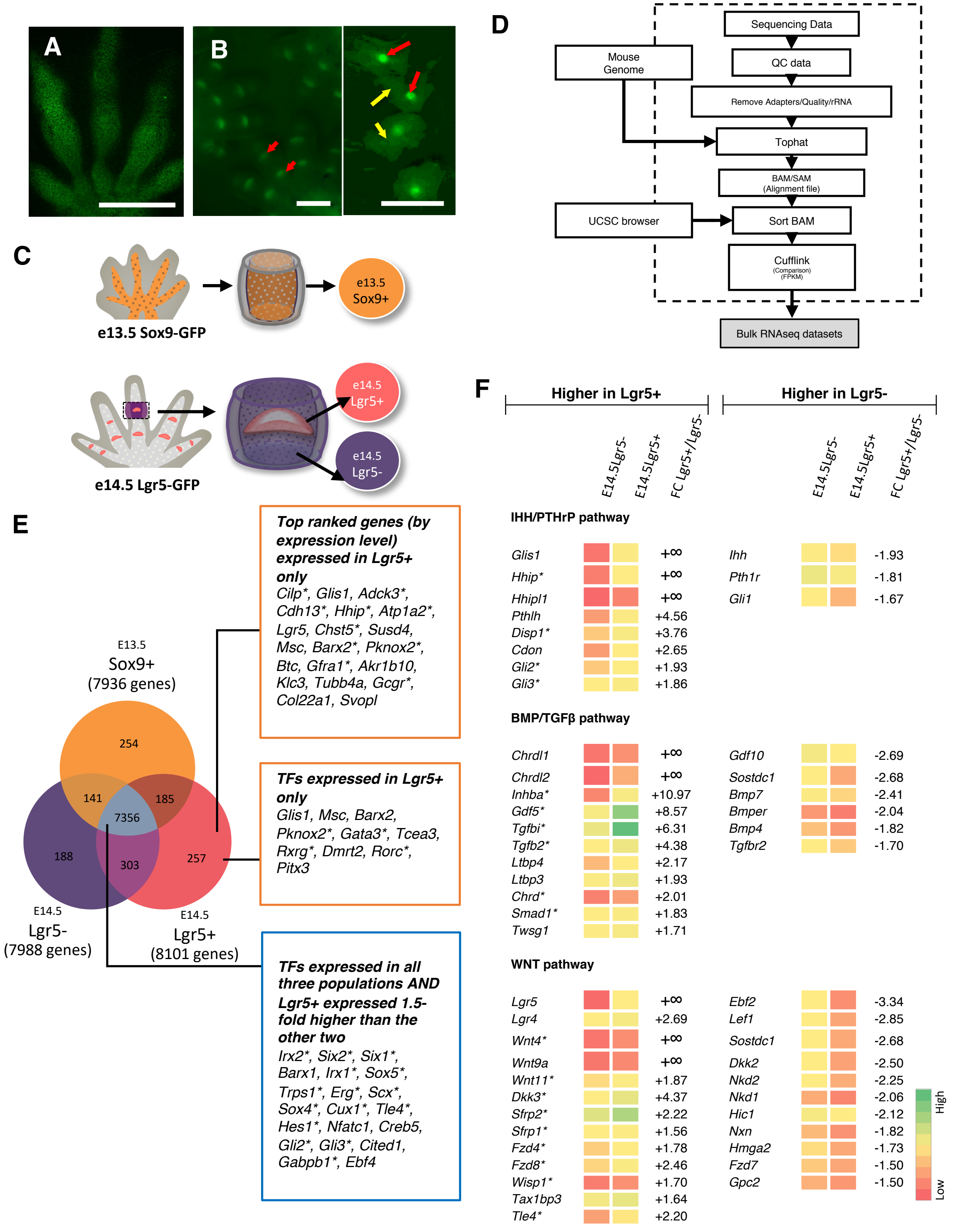

Figure S3. Bulk transcriptomic analysis of cell populations in joint development.
(A) Three population of cells isolated. One from Sox9 expressing cells in E13.5 digit of Sox9-eGFP mice, and two populations from the E14.5 interzone region of the developing digits that contains the Lgr5+ interzone (red arrows) and surrounding non-GFP region (yellow arrows) from Lgr5-GFP mice **(B)**. Scalebars, 1mm. **(C)** Diagrammatic illustration of the protocol with all cell isolated were sorted for the appropriate population of cells for subsequent for RNA-seq for the generating the three datasets. **(D)** Workflow for bulk RNAseq data processing. **(E)** Venn diagram showing the number of genes identified, and overlaps between datasets. Genes with higher expression in Lgr5+ population are shown on the right. **(F)** Heat maps depicting comparative gene expression levels of the top genes involved in specified molecular pathways. + Fold change (FC) indicates an increase and -FC for decrease in Lgr5+ cells. "+∞ " are given to indicate genes that are expressed only in Lgr5+ cells. Genes with expression data available in the Eurexpress database are marked with an asterisk.
Related to figure 3.

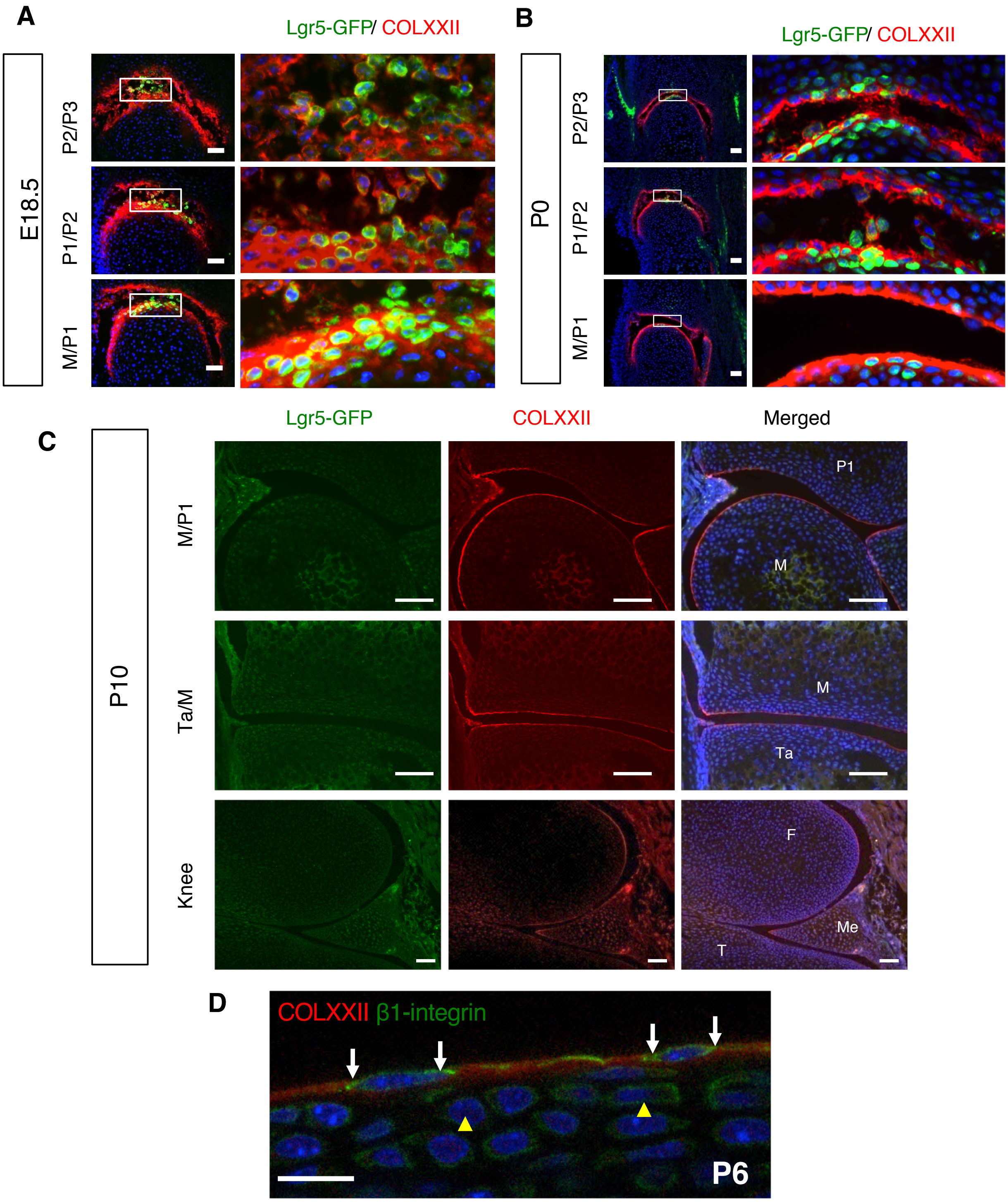

Figure S4. Expression of Lgr5 and COLXXII in phalangeal and knee joints.

Co-localization of Lgr5 (Green) and COLXXII (Red) in E18.5 **(A)** and newborn (P0) **(B)** digit joints. **(C)** Expression in M/P1, Ta/M and Knee joints at P10. M, metatarsal. P1, proximal phalange. P2, middle phalange. P3, distal phalange. Ta/M, tarsometatarsal joint. **(D)** Cells embedded in the COLXXII layer are flat compare to the round chondrocytes in the deeper zones (yellow arrow) of P6 mouse. Focal adhesions (β1-integrin) are found at both sides of the cells where they are in direct contact with the COLXXII matrix (white arrows). Scale bars in (A-C), 100μm and in (D), 10μm. **Related to figure 3.**

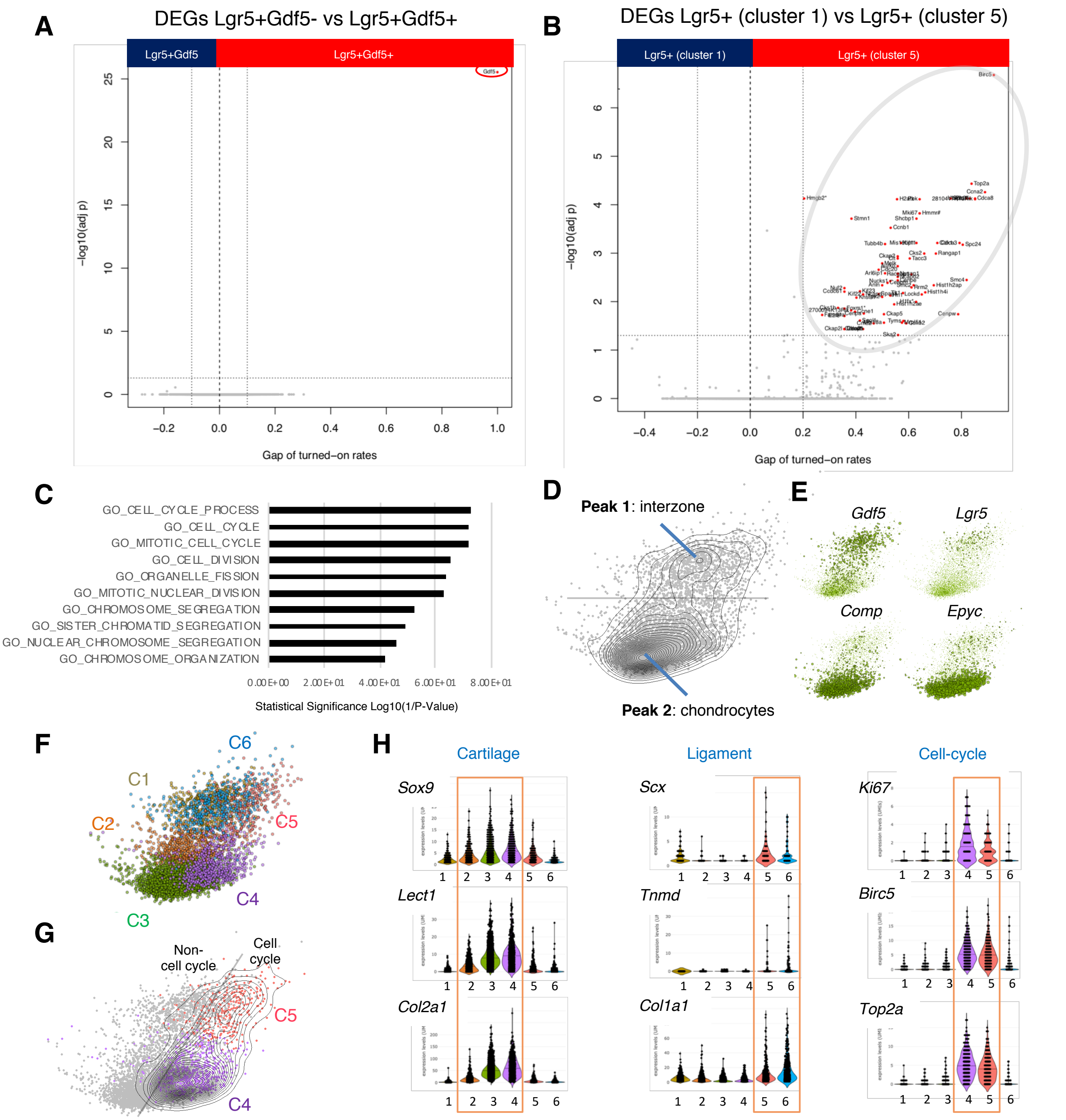

Figure S5 Additional information for single cell analysis

(A) Volcano plot showing *Gdf5* (red circle) is the only differential expressing gene (DEG) between *Lgr5+/Gdf5-* and *Lgr5+/Gdf5+* subpopulations, and these cells are essential identical. **(B)** Volcano plot shows DEGs between Lgr5+ cells from cluster 1 and cluster 5, also show they are the same cells, differ only as proliferating cells in cluster 5. **(C)** GO term analysis of the DEGs from panel (B) (circled). (d-g) Principle component analysis (PCA) for the 5,460 E14.5 knee joint cells. **(D)** A contour plot showing the density of cells, two major peaks are found. **(E)** Peak 1 corresponds to interzone cells expressing *Gdf5* and *Lgr5*, and peak 2 as non-interzone expressing mature chondrocyte markers, *Comp* and *Epyc*. **(F)** t-SNE clusters 1, 2, 5, and 6 are located in peak 1, whereas clusters 3 and 4 are in peak 2, consistent with the 2D t-SNE finding. **(G)** Contour plot of the cell cycle genes showing the existence of proliferating subpopulation of cell in the interzone (cluster 5) and non-interzone (cluster 4) clusters. **(H)** Violin plots showing the expression profiles of representative cartilage, ligament and cell cycle genes in each cluster supporting their identities/signatures. **Related to figure 4.**

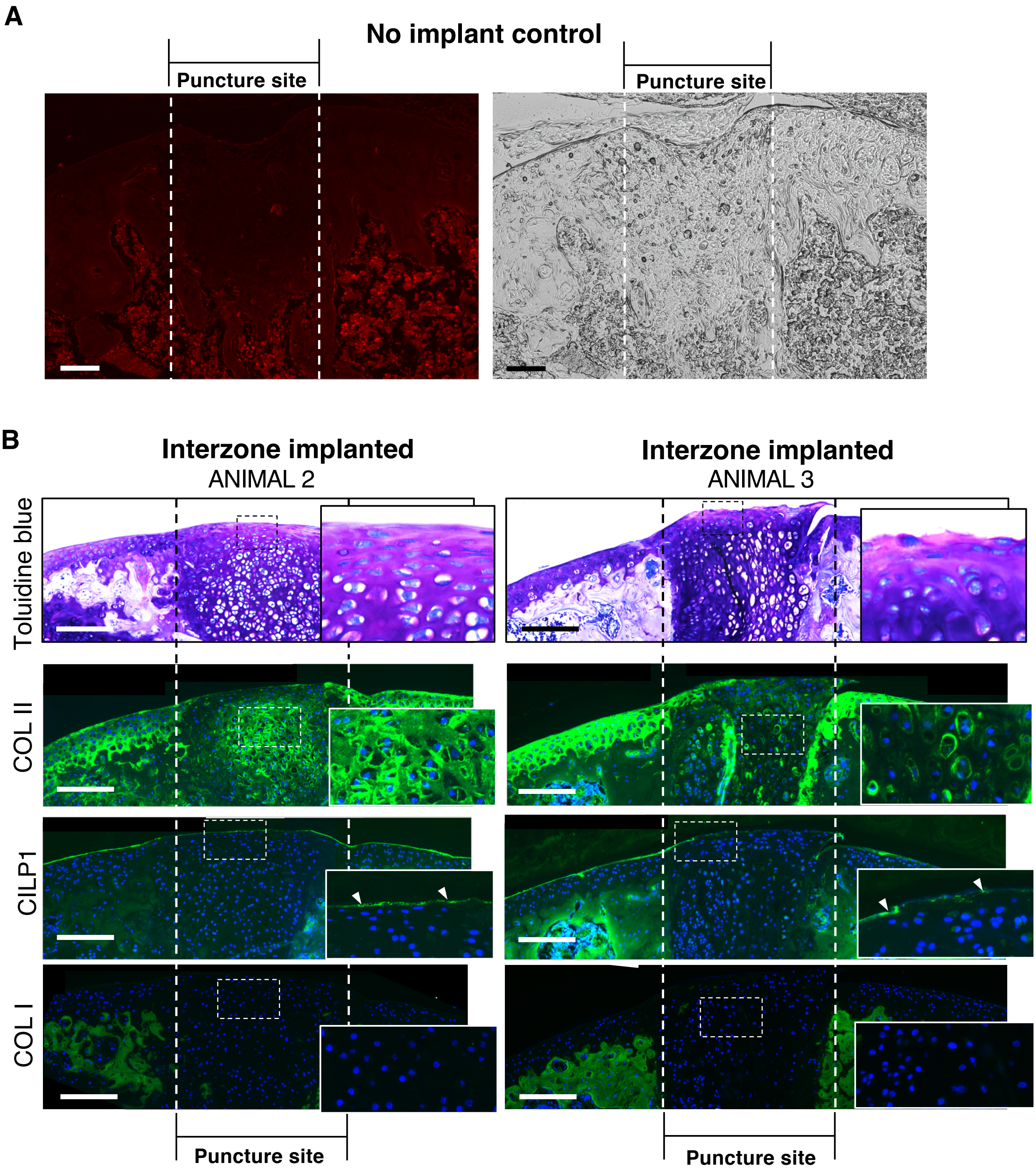

Figure S6. *Lgr5*+ interzone tissue transplantation for repairing of a cartilage lesion
(A) No tdTomato+ cells was detected in the puncture site without interzone tissue insert. Bright-field image of the same region was shown on the right. **(B)** Analysis of two additional animals receiving interzone tissue transplantation. Similar positive outcomes among all three operated animals, with differentiation of the interzones to chondrocytes expressing COL II, reestablishment of the superficial layer (CILP1 expressing), and suppressing COL I expression and fibrotic tissue. Scalebars, 50μm. **Related to figure 6.**

Table S1. Genes expressed by only one population (top 100 ranked by level) (Digit bulk)

	Expressed in Sox9+ only (FPKM>5)				Expressed in Lgr5- only (FPKM>5)				Expressed in Lgr5+ only (FPKM>5)			
	Gene	e13.5 Sox9+	e14.5 Lgr5-	e14.5 Lgr5+	Gene	e13.5 Sox9+	e14.5 Lgr5-	e14.5 Lgr5+	Gene	e13.5 Sox9+	e14.5 Lgr5-	e14.5 Lgr5+
**1**	*Epyc*	**48.82**	0.63	1.2	*Alas2*	0.86	**25.41**	1.16	*Cilp*	0.2	0.75	**58.03**
**2**	*Smpd3*	**30.66**	0.29	0.19	*Foxf2*	2.52	**20.2**	2.85	*Glis1*	4.33	3.54	**52.57**
**3**	*Panx3*	**27.67**	1.16	1.14	*Stra6*	4.58	**18.26**	4.13	*Adck3*	0.99	2.62	**35.46**
**4**	*Epha2*	**20.86**	4.24	4.68	*Cpa3*	0.81	**15.64**	2.79	*Cdh13*	2.91	2.97	**35.39**
**5**	*Frzb*	**20.82**	1.77	2.12	*Sst*	0.23	**15.56**	4.07	*Hhip*	2.97	4.94	**30.22**
**6**	*Foxd1*	**19.47**	3.43	0.71	*Cd74*	1.82	**12.69**	4.52	*Atp1a2*	3.4	1.89	**27.54**
**7**	*Cxcl10*	**18.67**	1.73	3.35	*Zfhx4*	4.26	**12.46**	4.66	*Lgr5*	3.48	1.36	**26.75**
**8**	*Pltp*	**17.52**	3.89	2.93	*Cma1*	0.39	**12.41**	2.26	*Chst5*	4.06	3.01	**26.54**
**9**	*Hoxc10*	**17.02**	0.21	0.37	*Ablim1*	3.74	**12.18**	4.9	*Susd4*	3.6	4.89	**22.94**
**10**	*Tnfaip3*	**16.98**	4.13	4.71	*S100a8*	0	**11.5**	0.86	*Msc*	2.18	1.28	**22.79**
**11**	*Peg10*	**14.87**	3.71	2.4	*Grp*	1.24	**11.19**	4.11	*Barx2*	1.27	0.25	**20.41**
**12**	*Spp1*	**14.72**	2.5	2.13	*Snca*	0.69	**9.95**	1.52	*Pknox2*	3.74	1.42	**19.37**
**13**	*Sgms2*	**14.54**	3.67	3.36	*Fgf2*	4.52	**9.61**	4.19	*Btc*	0.46	0.56	**19.22**
**14**	*Chac1*	**14.27**	0.64	1.38	*Angpt2*	0.78	**9.45**	1.97	*Gfra1*	2.9	3.67	**18.25**
**15**	*Ndufa4l2*	**14.12**	2.13	3.35	*Kcp*	4.12	**9.03**	4.84	*Akr1b10*	3.87	1.78	**18.06**
**16**	*Chst11*	**14.06**	4.4	4.75	*Arhgdig*	4.34	**9.01**	4.21	*Klc3*	3.59	4.05	**17.07**
**17**	*Ptgs2*	**13.6**	1.04	1.41	*Axin2*	2.12	**8.94**	3.13	*Tubb4a*	4.29	4.63	**16.12**
**18**	*Mpzl2*	**13.06**	3.79	4.67	*Cd52*	0	**8.86**	2.87	*Gcgr*	3.2	1.77	**15.97**
**19**	*Edil3*	**12.86**	3.73	4.66	*Kcnk2*	3.13	**8.79**	4.11	*Col22a1*	1.5	1.93	**15.82**
**20**	*S100b*	**12.1**	2.56	2.62	*Stmn2*	2.71	**8.7**	1.51	*Svopl*	1.11	1	**15.72**
**21**	*Foxo6*	**12.07**	4.31	3.36	*Dbx2*	1.4	**8.68**	0.74	*Vaultrc5*	4.63	2.57	**15.48**
**22**	*Sytl2*	**12.03**	2.32	4.18	*Alox5ap*	1.35	**8.64**	2.61	*Pde2a*	2.11	2.81	**15.23**
**23**	*C1galt1*	**11.68**	3.47	4.69	*Nxnl2*	2.17	**8.62**	3.73	*Ppp1r1b*	2	2.62	**14.44**
**24**	*Cgref1*	**11.62**	4.64	4.25	*Srgn*	0.45	**8.58**	2.88	*Sntb1*	1.77	2.9	**13.36**
**25**	*Pmaip1*	**11.61**	2.98	2.64	*Cmpk2*	3.86	**8.56**	4.93	*Thbs4*	2.09	2.94	**12.9**
**26**	*Tcf15*	**11.41**	4.13	3.25	*F11r*	3.94	**8.27**	3.13	*Krt5*	4.61	1.24	**12.62**
**27**	*Saa1*	**11.33**	2.08	4.06	*Kif26a*	3.06	**8.07**	1.13	*Lrrn3*	2.5	1.52	**12.38**
**28**	*Hck*	**10.72**	4.42	1.37	*Gstm6*	2.7	**8.06**	3.74	*Tnnc1*	1.83	1.07	**12.27**
**29**	*Xylt1*	**10.7**	4.44	3.26	*Heph*	2.99	**7.92**	3.35	*Ephx1*	1.74	2.72	**12.15**
**30**	*Entpd6*	**10.53**	4.55	4.95	*H2-T22*	2.59	**7.91**	4.31	*Stc1*	3.61	4.5	**11.55**
**31**	*Col15a1*	**10.4**	3.93	2.15	*Il17rd*	2.63	**7.89**	2.76	*Cer1*	2.53	0.03	**11.04**
**32**	*Magel2*	**10.39**	4.97	3.5	*Gpr124*	4.32	**7.8**	2.74	*Ptpro*	0.68	0.76	**10.97**
**33**	*Ifitm5*	**10.38**	2.22	1.74	*Fbxl19*	4.86	**7.78**	4.63	*Matk*	2.46	2.32	**10.56**
**34**	*Bmp5*	**10.25**	4.5	2.69	*Tmtc2*	2.81	**7.77**	2.37	*Acta2*	2.64	3.18	**10.21**
**35**	*E2f7*	**9.81**	4.32	3.38	*Lgals7*	2.52	**7.75**	3.95	*Ppp1r1a*	3.75	2.33	**9.98**
**36**	*Foxa3*	**9.6**	2.47	3.36	*Raver1-fdx1l*	3.61	**7.36**	3.59	*Kcnma1*	4.52	2.85	**9.71**
**37**	*Ssfa2*	**9.58**	4.32	4.96	*Adamts9*	3.79	**7.33**	3.19	*Sh2b2*	3.44	4.96	**9.6**
**38**	*Clec11a*	**9.43**	2.23	1.37	*Nek8*	4.51	**7.31**	4.84	*Glrb*	2.27	4.98	**9.5**
**39**	*Eif3j*	**9.34**	0.33	0.66	*Hdc*	0.85	**7.31**	0.71	*Des*	1.17	1.61	**9.33**
**40**	*Serinc5*	**9.13**	2.57	2.94	*Ngfr*	0.67	**7.28**	3.72	*Camk1g*	3.22	4.51	**9.32**
**41**	*Ncoa7*	**9.03**	2.27	2.52	*Scara5*	1.44	**7.15**	3.18	*Pitpnm1*	4.26	4.74	**9.26**
**42**	*Hoxa5*	**8.96**	2.36	1.97	*Pdxk*	4.82	**7.12**	4.5	*Gdf1*	3.37	3.67	**9.1**
**43**	*Dlc1*	**8.89**	3.62	3.41	*Kctd1*	4.39	**7.12**	3.9	*Krtdap*	0.62	0.19	**9.02**
**44**	*E2f8*	**8.67**	2.56	3.61	*Cav1*	2.19	**6.92**	3.6	*Kcnj15*	0.4	0.47	**9.02**
**45**	*Otor*	**8.65**	3.95	2.44	*Tubb3*	2.06	**6.9**	1.24	*Ccl4*	1.41	4.75	**8.88**
**46**	*Stil*	**8.53**	3.47	3.27	*Arhgap39*	4.5	**6.89**	3.82	*Sfn*	3.54	1.38	**8.86**
**47**	*Avpr1a*	**8.45**	1.25	0.8	*Lypd6*	2.57	**6.71**	3.31	*Sema3e*	3.21	3.2	**8.8**
**48**	*Fndc3c1*	**8.39**	2.26	2.93	*Lbh*	4.96	**6.7**	2.52	*Myod1*	0.46	1.69	**8.77**

**49**	*Frat1*	**8.29**	2.48	4.36	*Slc25a14*	3.08	**6.7**	4.78	*Smtnl2*	3.1	4.07	**8.64**
**50**	*Armcx5*	**8.25**	3.75	4.53	*Ophn1*	4.37	**6.68**	4.33	*Pf4*	1.69	3.34	**8.57**
**51**	*Nol12*	**8.23**	4.59	4.21	*Adamts17*	3.42	**6.67**	3.66	*Snap91*	2.29	1.93	**8.43**
**52**	*Ptger4*	**8.16**	2.88	2.04	*Mterfd3*	3.9	**6.62**	4.78	*Dcx*	2.93	2.18	**8.41**
**53**	*Ttll3*	**8.13**	4.77	4.21	*Rev3l*	4.38	**6.58**	4.67	*Fam159a*	3.68	3.42	**8.39**
**54**	*Dclre1c*	**8.02**	2.77	4.02	*Acsf3*	3.84	**6.54**	3.97	*Accs*	2.66	3.37	**8.32**
**55**	*Stk17b*	**8**	2.3	1.7	*Stom*	2.8	**6.54**	4.95	*Gata3*	3.49	0.84	**8.3**
**56**	*Hist1h2bn*	**8**	3.32	3.49	*Ank2*	3.39	**6.48**	4.86	*Myl1*	1.83	2.24	**8.28**
**57**	*Omd*	**7.96**	3.38	3.72	*Bcl2l11*	3.94	**6.42**	4.94	*Slc25a23*	2.77	4.17	**8.13**
**58**	*Pcdh1*	**7.9**	0.67	2.57	*Ankrd44*	2.78	**6.42**	3.49	*Tcea3*	1.68	4.53	**8.08**
**59**	*Npat*	**7.88**	4.84	4.74	*Nkain1*	3.17	**6.4**	3.93	*Doc2g*	4.83	4.62	**7.9**
**60**	*Cbr2*	**7.86**	4.12	3.25	*Ptges*	3.93	**6.39**	3.44	*Spon2*	0.21	2.12	**7.89**
**61**	*Hspg2*	**7.84**	3.74	2.28	*Kit*	2.35	**6.38**	2.5	*Abcg4*	3.87	4.93	**7.88**
**62**	*Pygl*	**7.81**	3.93	3.2	*Mgat3*	3.77	**6.35**	3.81	*Adamts14*	3.42	2.32	**7.81**
**63**	*Krtcap3*	**7.75**	2.34	4.88	*Fzd10*	1.6	**6.33**	1.17	*Krt15*	2.78	1.78	**7.81**
**64**	*Shroom3*	**7.72**	4.68	4.46	*Slitrk5*	3.45	**6.31**	3.4	*Dleu2*	1.11	3.16	**7.77**
**65**	*Hist1h3g*	**7.67**	4.3	2.68	*Ddx23*	4.78	**6.29**	4.63	*Cntfr*	2.19	1.1	**7.71**
**66**	*Scd1*	**7.62**	3.42	1.66	*Camk2g*	4.35	**6.26**	4.27	*Phactr1*	1.35	1.55	**7.69**
**67**	*Ung*	**7.59**	3.32	2.78	*Fap*	1.5	**6.26**	3.85	*Fbp2*	4.11	1.92	**7.65**
**68**	*Nppc*	**7.59**	3.69	3.88	*Rhobtb2*	4.82	**6.25**	4.68	*Ano1*	2.91	2.32	**7.62**
**69**	*Baiap2l1*	**7.58**	2.65	2.78	*Tgfbr3*	4.87	**6.22**	3.44	*Ehbp1l1*	3.16	3.34	**7.59**
**70**	*Tgfa*	**7.57**	2.66	1.99	*Atxn7l1*	4.59	**6.19**	3.89	*Uap1l1*	3.42	4.25	**7.53**
**71**	*Adamts3*	**7.53**	4.01	2.9	*Wbp7*	4.39	**6.12**	4.97	*Wdr67*	4.62	4.69	**7.41**
**72**	*Prkg2*	**7.48**	1.73	1.22	*Iqgap2*	4.99	**6.1**	4.34	*Tmem117*	1.95	1.4	**7.39**
**73**	*Orc1*	**7.4**	3.33	3.57	*Sdpr*	2.35	**6.09**	3.2	*Dynlt3*	4.2	4.23	**7.38**
**74**	*Aldoc*	**7.38**	3.34	4.4	*Zfp322a*	4.84	**6.06**	4.9	*Tle6*	3.26	4.42	**7.36**
**75**	*Foxf1a*	**7.37**	0.58	1.26	*Slc2a4*	3.46	**6.06**	2.19	*Ramp1*	0.56	0.62	**7.33**
**76**	*Cenpk*	**7.36**	4.04	4.36	*Pear1*	4.79	**6.04**	3.57	*Rxrg*	4.78	2.31	**7.22**
**77**	*Fam180a*	**7.35**	1.15	2.41	*Mtss1*	3.04	**6.03**	4.92	*St7l*	4.32	4.72	**7.18**
**78**	*Tgm2*	**7.34**	3.85	3.84	*Traf5*	4.46	**6.01**	4.74	*Wnt9a*	3.13	3.77	**7.17**
**79**	*Esco2*	**7.34**	4.01	4.35	*Fgf10*	2.45	**6**	2.21	*Tesk2*	4.7	4.25	**7.16**
**80**	*Hip1r*	**7.33**	3.6	4.68	*Gdf11*	3.4	**5.98**	4.88	*Coq10a*	4.14	4.78	**7.16**
**81**	*Slc19a2*	**7.32**	4.28	4.71	*Txnrd2*	3.51	**5.95**	3.92	*Fam20c*	3.93	3.24	**7.16**
**82**	*Cenpf*	**7.32**	3.48	4.93	*Tspan11*	1.58	**5.95**	3.57	*Rarres2*	3.09	3.66	**7.16**
**83**	*Pdlim1*	**7.29**	3.41	2.27	*Kpna6*	4.89	**5.94**	4.26	*Pip4k2a*	3.77	3.38	**7.12**
**84**	*Per2*	**7.28**	2.93	4.73	*Fert2*	4.55	**5.9**	4.66	*Dmrt2*	1.31	2.73	**7.06**
**85**	*Ascc3*	**7.27**	4.03	4.96	*Zfp397*	4.26	**5.9**	4.14	*Nt5e*	2.7	1.32	**7.02**
**86**	*St6galnac2*	**7.25**	4.83	3.19	*Smcr8*	4.69	**5.89**	3.4	*Tagln*	3.33	2.95	**7.01**
**87**	*Scn1b*	**7.22**	2.19	2.08	*Gng8*	3.13	**5.88**	3.46	*Col13a1*	0.75	4.35	**6.98**
**88**	*Grem1*	**7.16**	1.11	2.71	*Sall1*	2.8	**5.88**	2.77	*Aard*	3.16	3.38	**6.97**
**89**	*Nptx2*	**7.12**	1.28	0.73	*Nlrx1*	2.62	**5.87**	4.8	*Tesc*	1.57	0.78	**6.95**
**90**	*Usp2*	**6.93**	4.18	3.64	*Prmt10*	4.21	**5.86**	4.73	*Shq1*	4.9	4.87	**6.91**
**91**	*Gpd2*	**6.93**	4.65	4.22	*Tcf7*	2.91	**5.86**	3.27	*Ell3*	3.03	2.34	**6.91**
**92**	*Snhg4*	**6.91**	3.3	2.85	*Slc22a5*	3.73	**5.84**	4.4	*C1qa*	0.31	0.86	**6.91**
**93**	*Isl1*	**6.86**	0.14	0.02	*Ptch2*	2.46	**5.83**	1.86	*Tmem175*	4.76	4.96	**6.87**
**94**	*Lingo1*	**6.78**	3.68	2.82	*Prr5l*	1.38	**5.83**	1.75	*Dner*	0.57	1.67	**6.83**
**95**	*Pcp4*	**6.75**	2.28	1.82	*Tyk2*	4.38	**5.79**	4.23	*Apba2*	3.22	3.57	**6.81**
**96**	*Fam84b*	**6.75**	2.9	3.26	*Cdc42bpa*	4.25	**5.75**	4.38	*Ppp1r3g*	3.54	1.43	**6.79**
**97**	*Dlx3*	**6.69**	3.11	1.26	*Srd5a1*	4.44	**5.73**	4.28	*Dcaf6*	3.38	3.82	**6.78**
**98**	*Rfx1*	**6.68**	4.95	3.69	*Ccdc28a*	3.3	**5.73**	3.59	*Igsf11*	2.7	1.46	**6.77**
**99**	*Arl4c*	**6.67**	2.97	3.03	*Pcnx*	4.42	**5.72**	4.81	*Krt14*	4.43	0.54	**6.76**
**100**	*Foxn2*	**6.64**	3.72	4.97	*Hnrnpul2*	4.27	**5.69**	3.96	*Fbp1*	1.5	1.46	**6.76**

Table S2. Transcription factors expressed in specific cell population (Bulk transcriptome of digit joint)

Gene	e13.5 Sox9+	e14.5 Lgr5- (FPKM)	e14.5 Lgr5+
**Transcription factors expressed in Lgr5+ only**			
Glis1	4.33	3.54	**52.57**
Msc	2.18	1.28	**22.79**
Barx2	1.27	0.25	**20.41**
Pknox2	3.74	1.42	**19.37**
Gata3	3.49	0.84	**8.3**
Tcea3	1.68	4.53	**8.08**
Rxrg	4.78	2.31	**7.22**
Dmrt2	1.31	2.73	**7.06**
Rorc	1.03	0.62	**6.02**
Pitx3	0.79	1.01	**5.71**
**Transcription factors expressed in Lgr5- only**			
Foxf2	2.52	**20.2**	2.85
Dbx2	1.4	**8.68**	0.74
Lbh	4.96	**6.7**	2.52
Ptges	3.93	**6.39**	3.44
Wbp7	4.39	**6.12**	4.97
Rfx5	4.76	**5.54**	4
Tcf20	3.21	**5.48**	3.03
**Transcription factors expressed in Sox9+ only**			
Foxd1	**19.47**	3.43	0.71
Hoxc10	**17.02**	0.21	0.37
Tnfaip3	**16.98**	4.13	4.71
Foxo6	**12.07**	4.31	3.36
Tcf15	**11.41**	4.13	3.25
Foxa3	**9.6**	2.47	3.36
Hoxa5	**8.96**	2.36	1.97
Foxf1a	**7.37**	0.58	1.26
Pdlim1	**7.29**	3.41	2.27
Per2	**7.28**	2.93	4.73
Rfx1	**6.68**	4.95	3.69
Foxn2	**6.64**	3.72	4.97
Pou3f3	**6.58**	1.83	2.45
Zim1	**6.44**	1.13	1.16
Per1	**6.41**	3.16	2.64
Hoxc9	**6.32**	0.06	0.34
Meox1	**6.03**	1.63	3.26
Nkx6-2	**5.41**	4.07	4

Table S3. Transcription factors expressed in all three populations AND Lgr5+ expressed 1.5-fold higher than the other two populations (Bulk transcriptome of digit joint)

Gene	e13.5 Sox9+	e14.5 Lgr5- (FPKM)	e14.5 Lgr5+	Fold change Lgr5+/Lgr5-	Fold change Lgr5+/Sox9+
Irx2	9.97	5.84	57.21	+9.80	+5.74
Six2	50.34	53.86	277.21	+5.15	+5.51
Six1	16.24	9.49	37.33	+3.93	+2.30
Barx1	55.41	23.81	92.04	+3.87	+1.66
Irx1	15.6	11.76	43.98	+3.74	+2.82
Sox5	21.32	10.02	35.08	+3.50	+1.65
Trps1	15.71	12.68	33.33	+2.63	+2.12
Erg	10.34	9.99	25.21	+2.52	+2.44
Scx	42.52	80.56	194.54	+2.41	+4.58
Sox4	416.76	318.43	701.26	+2.20	+1.68
Cux1	47.5	33.43	73.42	+2.20	+1.55
Tle4	14.27	11.65	25.58	+2.20	+1.79
Hes1	298.41	284.69	604.86	+2.12	+2.03
Nfatc1	52.48	39.2	80.79	+2.06	+1.54
Creb5	19.1	25.8	49.93	+1.94	+2.61
Gli2	13	19.73	38.16	+1.93	+2.94
Gli3	22.69	32.62	60.75	+1.86	+2.68
Cited1	10.79	11.28	19.14	+1.70	+1.77
Gabpb1	30.91	29.95	50.67	+1.69	+1.64
Ebf4	7.67	11.14	18.09	+1.62	+2.36

Table S4. Genes involved in molecular signaling pathways, cell-matrix contact, ECM degradation and organisation (Bulk transcriptome of E14.5 digit joint)

Gene	E14.5 Lgr5- (FPKM)	E14.5 Lgr5+ (FPKM)	Fold change relative to Lgr5-
**IHH/PTHrP pathway**			
Glis1	3.54	52.57	+∞
Hhip	4.94	30.22	+∞
Hhipl1	0.92	6.36	+∞
Pthlh	11	50.11	+4.56
Disp1	19.78	74.28	+3.76
Cdon	10.93	28.96	+2.65
Gli2	19.73	38.16	+1.93
Gli3	32.62	60.75	+1.86
Ihh	45.36	23.54	-1.93
Pth1r	131.09	72.3	-1.81
Gli1	26.77	16.03	-1.67
**BMP/TGFβ pathway**			
Chrdl1	3.52	8.64	+∞
Chrdl2	0.85	14.32	+∞
Inhba	6.76	74.18	+10.97
Gdf5	71.72	614.83	+8.57
Tgfbi	136.76	863.5	+6.31
Tgfb2	27.44	120.3	+4.38
Ltbp4	16.72	36.21	+2.17
Ltbp3	61.32	118.37	+1.93
Chrd	5.69	11.43	+2.01
Smad1	32.44	59.33	+1.83
Twsg1	43.31	73.92	+1.71
Gdf10	86.28	32.02	-2.69
Sostdc1	36.56	13.65	-2.68
Bmp7	52.52	21.81	-2.41
Bmper	10.35	5.08	-2.04
Bmp4	18.37	10.07	-1.82
Tgfbr2	33.63	19.82	-1.70
**WNT pathway**			
Lgr5	1.36	26.75	+∞
Lgr4	28.27	76.06	+2.69
Wnt4	3.52	8.09	+∞
Wnt9a	3.77	7.17	+∞
Wnt11	23.2	43.28	+1.87
Dkk3	37.72	164.84	+4.37
Sfrp2	203.06	450.71	+2.22
Sfrp1	28.5	44.32	+1.56
Fzd4	20.58	36.73	+1.78
Fzd8	21.71	53.42	+2.46
Wisp1	5.11	8.68	+1.70
Tax1bp3	92.74	151.9	+1.64
Tle4	11.65	25.58	+2.20
Ebf2	27.59	8.27	-3.34
Lef1	36.27	12.73	-2.85
Sostdc1	36.56	13.65	-2.68
Dkk2	30.03	11.99	-2.50
Nkd2	32.47	14.43	-2.25
Nkd1	13.91	6.74	-2.06
Hic1	78.08	36.77	-2.12
Nxn	16.76	9.19	-1.82
Hmga2	23.57	13.6	-1.73
Fzd7	19.6	13.08	-1.50
Gpc2	15.06	10.06	-1.50

Gene	E14.5 Lgr5- (FPKM)	E14.5 Lgr5+ (FPKM)	Fold change relative to Lgr5-
**Cell surface markers and receptors**			
Cd9	105	296.73	+2.83
Cd200	6.66	14.9	+2.24
Cd83	22.25	45.54	+2.05
Cd47	22.63	37.49	+1.66
Cd52	8.86	2.87	-∞
Cd74	12.69	4.52	-∞
Cd248	46.41	21.55	-2.15
Cd82	10.93	6.35	-1.72
Cdh13	2.97	35.39	+∞
Itga11	16.04	81.87	+5.10
Itga5	29.83	52.72	+1.77
Itgav	9.39	5.39	+1.37
Itga9	8.32	11.38	+1.05
Itgb1	92.38	97.13	+1.05
Itgb5	70.15	73.66	-2.02
Cdh3	33.83	16.78	-1.62
Cdh2	21.39	13.23	-1.76
Ddr2	15.06	8.55	-1.74
Itga6	5.78	5.44	-1.06
**Enzymes for ECM degradation**			
Adamts14	2.32	7.81	+∞
Adamtsl2	6.99	14.82	+2.12
Timp3	49.55	85.52	+1.73
Ctsf	24.52	36.84	+1.50
Chst10	10.7	6.78	-1.58
Chst15	7.46	3.17	-∞
Mmp11	124.76	77.61	-1.61
**ECM components**			
Cilp	0.75	58.03	+∞
Col22a1	1.93	15.82	+∞
Thbs4	2.94	12.9	+∞
Spon2	2.12	7.89	+∞
Tgfbi	136.76	863.5	+6.31
Fmod	24.45	134.56	+5.50
Col8a1	6.86	22.27	+3.25
Dcn	35.98	115.28	+3.20
Efemp1	12.14	34.69	+2.86
Col9a2	216.81	479.99	+2.21
Ltbp4	16.72	36.21	+2.17
Col9a3	206.32	440.71	+2.14
Col6a1	82.63	167.27	+2.02
Ltbp3	61.32	118.37	+1.93
Vcan	31.28	56.73	+1.81
Wisp1	5.11	8.68	+1.70
Col9a1	184.09	302.35	+1.64
Col11a1	104.96	169.86	+1.62
Col2a1	1400.84	2253.09	+1.61
Postn	52.87	81.99	+1.55
Emilin1	159.14	244.21	+1.53
Fbln2	159.5	243.39	+1.53
Prg4	2.36	1.14	not exp. in both
Comp	15.75	3.9	-∞
Emilin2	18.61	6.65	-2.80
Col23a1	25.86	10.96	-2.36
Matn1	93.79	41.46	-2.26
Fbn1	26.53	12.34	-2.15
Col4a1	50.96	28.98	-1.76
Fbln5	21.76	13.65	-1.59
Lox	47.87	31.36	-1.53

+∞ and -∞ indicate genes that are not expressed in Lgr5- and Lgr5+ respectively, fold change is not applicable.

Table S5. Details and parameters for single cell transcriptome sequencing and analysis. Related to Figures 4 and 5.

**Sequencing and quality check**	
Sequencing depth/read length	50Gbp/101bp pair end
Total read-pairs	304 million
Percentage achieving a quality score of Q30 (equivalent to 1 mistake in 1,000 bps)	72%
Mapping rate (to mouse reference mm10)	93.0%
Percentage of valid barcodes	97.7%
Sequencing saturation	77.2%
Fraction of reads in cells and beads	100%
Cells	*90.9%*
Background beads	*9.1%*
**Transcriptome mapping**	
Total number of genes detected in aggregate	18,247
Total number of UMIs detected	46,239,500
Number of reads (R2) per UMI detected	6.6 (=304million/46million)
Total number of cells captured and sequenced	5,649
Average number of gene detected	2,058 (s.d. 637 genes)
Number of UMIs per cell	6,601
Blood cell captured	189
Average number of genes detected in blood cell	216
Non-blood cells	5,460 (=5649-189)
Total number of Lgr5+ cell detected	207
Express normal Lgr5 allele only	*94*
Express Lgr5-CRE-ERT2 allele only	*87*
Express both alleles	*26*
**Data analysis**	
DEG stringency	• FDR cutoff of <0.05.<br>• expression difference at least 20% (defined as %{turned on in cluster_i} - %{turned on in all_other_clusters})
Clustering of dispersed genes (Heatmap)	• Top 5% dispersed gene (genome-wide)<br>• at least 50 cells expressed
Pseudotime trajectory	• Negative binomial distribution<br>• based on the genome-wide UMI count data for genes expressing in 10 cells or more

Table S6. DEGs "Cluster 1 vs non-cluster 1" and "Lgr5+ vs Lgr5-" (Single cell transcriptome of E14.5 knee joint). Related to Figure 4

<table>
<tr>
<td rowspan="3">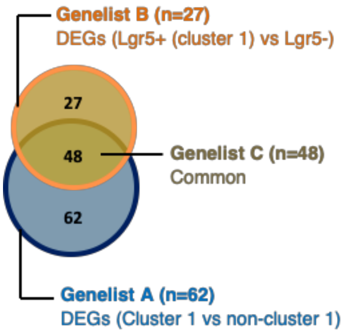
</td>
<td>Genelist A (n=62)
Osr2, Man1a, Nbl1, Angptl2, Dusp1, Egfl6, Gas2, Rbms3, Creb5, Rnd3, Zfp503, Dclk1, F2r, Zfp36l1, Igfbp4, 3632451o06rik, Epb41l3, Svil, Tpm1, Palld, Tuba1a, Hmcn1, Cdh11, Hspa1a, Pdgfra, Cpq, Epha7, Foxp1, Loxl2, Csrp1, Dbn1, Col6a1, Col8a2, Vcan, Ddah2, Col6a2, Cdon, Prrx1, Tagln2, Sulf2, Col5a2, Tax1bp3, Zyx, Nrep, Ltbp3, Nfia, Rcan1, Fbln1, Igfbp7, Arhgap31, Marcksl1, Kctd12, Hoxa10, Abracl, Marcks, Hmgb3, Timp2, Map1lc3a, Ifitm3, Aes, Trps1, Barx1</td>
</tr>
<tr>
<td>Genelist B (n=27)
F2rl1, Fhod3, Adam33, Tagln, Fst, Khdrbs2, Hspb8, Chst5, Inhba, Tnni2, Etl4, Meox2, Sorcs2, Tlr2, Bmp4, Gfra4, Syt11, Mafb, Ppm1l, Pdgfrl, Gpm6b, Fjx1, Tmem119, Pcdh18, Enpp1, Rgcc, Pdgfc</td>
</tr>
<tr>
<td>Genelist C (n=48)
Lrrc17, Pamr1, Htra1, Cdh13, Phactr1, Aqp1, Tm4sf1, Nupr1, Tcea3, Lrrn1, Sfrp2, Ccdc80, Cntfr, Tgfbi, Ssc5d, Gas1, Pax9, Mkx, Pid1, Map1b, Gdf5, Dkk3, Sulf1, Gm26771, Dusp8, Mylk, Shisa2, Adamtsl1, Prss23, Krt17, Cited2, Wisp1, Loxl1, Sox4, Lmo1, Ackr3, Bcl11a, Scx, Phldb2, Myo1b, Spint2, Fbn2, Postn, Mfap4, Col12a1, Tgfb2, Fn1, Ier3</td>
</tr>
</table>

Bold = transcription factors

Table S7. Primers used in this paper

	Forward primer	Reverse primer
**Primers for genotyping Lgr5-eGFP-IRES-CreER^{T2} mice**		
wildtype	5'-CTGCTCTCTGCTCCCAGTCT-3'	5'-ATACCCCATCCCTTTTGAGC-3'
mutant		5'-GAACTTCAGGGTCAGCTTGC-3'
**Primers for genotyping R26R-lacZ mice**		
wildtype	5'-AAAGTCGCTCTGAGTTGTTAT-3'	5'-GGAGCGGGAGAAATGGATATG-3'
mutant		5'-GCGAAGAGTTTGTCCTCAACC-3'
**Primers for Quantitative PCR**		
Lgr4	5'-CACAACCTGTCTGATCTGCAT-3'	5'-TGGTGCTTATTTTTGTCCCTGT-3'
Lgr5	5'-CACAGCCACTGCGGCGACTT-3'	5'-CAATGGGCGTCTGCCGGGTC-3'
Lgr6	5'-CTCCTTGCTGGACAAGCTGA-3'	5'-TCTCAAAGAGGTGCTCGCAG-3'
Gdf5	5'-AAAGGGAGGTAACAGCAGCG-3'	5'-CCCATCCTTCTCCAAGGCAC-3'
Cd90	5'-TCTCCTGCTCTCAGTCTTGC-3'	5'-TATTCTCATGGCGGCAGTCC-3'
Cd105	5'-TAGCACCTTGTCCCAGGAAG-3'	5'-CAGTACAGAGGGCAGGACAA-3'
Notch1	5'-TGTGAGTCCAACCCTTGTGT-3'	5'-CGTTGATGTTGGTCTGGCAA-3'
Klf4	5'-AAAAGAACAGCCACCCACACTT-3'	5'-CCCAGTCACAGTGGTAAGGTTTC-3'
Gapdh	5'-TGCACCACCAACTGCTTAG-3'	5'-GATGCAGGGATGATGTTC-3'
