# Supplementary material for: Lgr5 and Col22a1 Mark Progenitor Cells in the Lineage toward Juvenile Articular Chondrocytes
Source: Stem Cell Reports. 2019 Sep 12;13(4):713–29. doi: 10.1016/j.stemcr.2019.08.006 (PMC6829785; doi:10.1016/j.stemcr.2019.08.006)
Supplement: Document S2. Article plus Supplemental Information [file mmc2.pdf]

Stem Cell Reports
Article
OPEN ACCESS

Lgr5 and *Col22a1* Mark Progenitor Cells in the Lineage toward Juvenile Articular Chondrocytes

Chen Feng,[1,7,8] Wilson Cheuk Wing Chan,[1,2,8] Yan Lam,[1,8] Xue Wang,[1] Peikai Chen,[1] Ben Niu,[1] Vivian Chor Wing Ng,[1,2] Jia Chi Yeo,[3] Sigmar Stricker,[4,5] Kathryn Song Eng Cheah,[1] Manuel Koch,[6] Stefan Mundlos,[5] Huck Hui Ng,[3] and Danny Chan[1,2,*]

[1]School of Biomedical Sciences, The University of Hong Kong, Faculty of Medicine Building, 21 Sassoon Road, Pokfulam, Hong Kong SAR, China
[2]The University of Hong Kong - Shenzhen Institute of Research and Innovation (HKU- SIRI), Hi-Tech Industrial Park, Nanshan, Shenzhen, China
[3]Genome Institute of Singapore, Singapore, Singapore
[4]Freie Universität Berlin, Institut für Chemie und Biochemie, Berlin, Germany
[5]Max Plank Institute for Molecular Genetics, Berlin, Germany
[6]Institute for Dental Research and Oral Musculoskeletal Biology, Center for Biochemistry, Medical Faculty, University of Cologne, Cologne, Germany
[7]Hebei Orthopedic Clinical Research Center, The Third Hospital of Hebei Medical University, Shijiazhuang, 050051 Hebei, China
[8]Co-first author
*Correspondence: chand@hku.hk
https://doi.org/10.1016/j.stemcr.2019.08.006

SUMMARY

The synovial joint forms from a pool of progenitor cells in the future region of the joint, the interzone. Expression of *Gdf5* and *Wnt9a* has been used to mark the earliest cellular processes in the formation of the interzone and the progenitor cells. However, lineage specification and progression toward the different tissues of the joint are not well understood. Here, by lineage-tracing studies we identify a population of *Lgr5*$^+$ interzone cells that contribute to the formation of cruciate ligaments, synovial membrane, and articular chondrocytes of the joint. This finding is supported by single-cell transcriptome analyses. We show that *Col22a1*, a marker of early articular chondrocytes, is co-expressed with *Lgr5*$^+$ cells prior to cavitation as an important lineage marker specifying the progression toward articular chondrocytes. *Lgr5*$^+$ cells contribute to the repair of a joint defect with the re-establishment of a *Col22a1*-expressing superficial layer.

INTRODUCTION

Skeletal movement is facilitated by synovial joints comprising articular cartilage encased in a capsule. Healthy articular cartilage minimizes friction and shock impacts, but wears with aging, and repair is inefficient (Hunter, 1995). Tissue repair involves the recruitment/activation of local progenitor cells, often recapitulating cellular differentiation similar to embryonic development. Synovial joints form through dedifferentiation of chondrocytes at the site of future joints (Craig et al., 1987), defined as "interzones," marked by early expression of *Gdf5* and *Wnt9a* (Guo et al., 2004; Hartmann and Tabin, 2001), with progenitor properties (Koyama et al., 2008; Kozhemyakina et al., 2015). Studies using *Gdf5*-Cre (Koyama et al., 2008; Rountree et al., 2004) and inducible *Gdf5*-CreER^{T2} mice (Decker et al., 2017; Shwartz et al., 2016) confirmed *Gdf5*-expressing cells contribute to the formation of articular chondrocytes and other joint-related cells. Furthermore, expansion of *Gdf5*$^+$ cells in the interzone can come from recruitment of surrounding *Sox9*$^+$/*Gdf5*-mesenchymal cells (Shwartz et al., 2016). Regional localization of *Gdf5*$^+$ cells (central or peripheral) in the interzone may influence differentiation of joint cell lineages (Decker et al., 2017). Thus, the origin, timing, and location of interzone cells may determine cell fate in the joint tissues.

Lgr5, a member of the G-protein-coupled receptor family and a Wnt signaling target gene, is a stem cell marker for highly proliferative progenitor cells in the small intestine, colon, hair follicle, mammary gland, and ovary (Leung et al., 2018). Collagen XXII (COLXXII), encoded by the *Col22a1* gene, is an extracellular matrix (ECM) protein localized at the articular cartilage-synovial fluid junction (Koch et al., 2004). Its function is not well understood. A role as a negative regulator of chondrocyte hypertrophy through interacting with β1-integrin was proposed (Zwolanek et al., 2014). Here, we identify a population of *Lgr5*-expressing cells in the interzone of developing joints that contribute to the formation of the articular cartilage, cruciate ligaments, and meniscus. We show that *Col22a1* is expressed by *Lgr5*$^+$ interzone cells prior to the cavitation process, supporting a lineage progression from *Lgr5*$^+$ interzone cells to *Lgr5*$^+$/*Col22a1*$^+$ double-positive cells as committed progenitors for *Col22a1*$^+$ juvenile chondrocytes at the articular surface.

RESULTS

Lgr5 as a Novel Marker for Distinct Cells in Developing Synovial Joints

As interzone cells are progenitor cells, we screened these cells with a panel of stem cell markers and detected *Lgr5*

This is an open access article under the CC BY license (http://creativecommons.org/licenses/by/4.0/).

expression by qRT-PCR (Figure S1). Using *GFP* expression in *Lgr5-eGFP-IRES-CreER*^{T2} (Lgr5-GFP) mice, we confirmed *Lgr5* as a marker of interzone cells. *Lgr5-GFP* is a null allele, with *GFP* expression replacing *Lgr5* (Barker et al., 2007). Mice heterozygous for this allele are normal and viable, while homozygous mice die perinatally (Barker et al., 2007). However, we observed no abnormalities in limb development or synovial joint formation in homozygotes (Figure S2). All analyses of *Lgr5/GFP* expression in synovial joints were carried out in mice heterozygous for this allele.

Digit joints develop proximodistally, providing information on progression. By whole-mount analysis of Lgr5-GFP mice, we detected GFP in digit joints from embryonic day 13.5 (E13.5) to E18.5 (Figure 1A). At E13.5, the proximal M/P1 joint is clearly positive for GFP, whereas the P1/P2 joints show only a faint signal and no signal for the P2/P3 joints (Figure 1A), which was confirmed by histological analysis (Figure 1B). In the M/P1 joint of digit III, signal can be detected at E13.5 as a "salt and pepper" pattern in cells of the interzone, which becomes more intense and uniformly distributed in the center of the interzone from E14.5. With cavitation, *Lgr5*$^+$ cells are detected in the flanking regions of future articular cartilage, and the intensity and number of expressing cells decrease substantially.

Lgr5 Expression Begins after *Gdf5* Expression in Digit Joint Formation

Gdf5 is a marker for interzone cells (Merino et al., 1999; Storm and Kingsley, 1999). We compared the expression of *Lgr5* with that of *Gdf5* in adjacent sections (Figures 1C–1F) in digit III. *Gdf5* is expressed in the P2/3 interzone, the last joint formed at E14.5 (Figure 1C), but not *Lgr5* (Figure 1C), indicating a later onset. Both *Gdf5* and *Lgr5* are expressed in the more proximal P1/P2 and M/P1 joints. Interestingly, *Lgr5* expression is localized to a subset of interzone cells central to the *Gdf5* expression margin of each joint (Figure 1D). At E16.5, just before cavitation, *Gdf5* expression persists in a region of the interzones in a distinct horseshoe shape (Figure 1E), with *Lgr5*$^+$ cells localized to the center of the horseshoe (Figure 1F, circle) with distinct *Gdf5*$^+$ flanking cells. With cavitation, the number of *Lgr5*$^+$ cells decreases, whereas some *Gdf5*$^+$ cells are maintained at the articular surface (Figure 1F). Thus, *Lgr5* marks a subset of *Gdf5*-expressing cells in the interzone with a distinct temporal and spatial pattern in joint development.

Lgr5 Expression in the Developing Knee Joint

The knee joint is more complex, with additional structures of the meniscus and cruciate ligaments. Specific *Lgr5* expression can be seen from whole-mount imaging at E16.5 (Figure 1G). We examined histological sections at the peripheral (Figure 1I) and central (Figure 1J) regions of the developing joint from E13.5 to E18.5 as indicated in Figure 1H. *Lgr5* is expressed as early as E13.5 in the interzone, before formation of the meniscus, articular cartilage, and cruciate ligaments. From E16.5, concomitant with early-stage cavitation and formation of the meniscus and cruciate ligaments, to maturation at E18.5, *Lgr5* expression becomes restricted and weaker at the future articular surfaces of the knee joint (Figure 1I, peripheral sections). However, at this stage, many *Lgr5*$^+$ cells become evident in the developing lateral and medial meniscus (Figure 1I, peripheral sections). These *Lgr5*$^+$ cells are localized to the "lip" or narrow regions of the meniscus. Postnatally, *Lgr5* expression diminishes with little or no detection in cells of the articular cartilage or the meniscus by day 10 (P10) (Figure S4C). Formation of the cruciate ligaments also starts within the interzone. Strong *Lgr5* expression is detected in cruciate ligaments (Figure 1J, central sections), throughout the length of the ligaments from the base at the insertion site and into the cartilage element (Figure 1J).

Lgr5$^+$ Interzone Cells Are Progenitors for Interior Structures of the Knee Joint

We used *Lgr5-eGFP-IRES-CreER*^{T2} mice to tag and trace *Lgr5*$^+$ cells in the developing joints. A single injection of tamoxifen at E13.5 into *Lgr5-GFP*; *Rosa26-LacZ* (R26R) pregnant mice showed β-galactosidase-labeled (LacZ$^+$) cells in the digit (Figure 2A) and knee (Figure 2B) joint interzones at E15.5. At E17.5, descendants of *Lgr5*$^+$ cells persisted at cavitation (Figure 2C), and at P21 digit joints showed *Lgr5*$^+$ descendants throughout the full thickness of the articular cartilage (Figure 2E). In the developing knee, descendants of *Lgr5*$^+$ cells can be detected at E17.5 near the surface of the articular cartilage (Figure 2D, blue arrows), the meniscus (Figure 2D, red arrows), intrapatellar fat pad (Figure 2D, green arrows), and the developing cruciate ligaments (Figure 2D, yellow arrows). They can also be detected when traced to P21 (Figure 2F). Interestingly, many more descendants of *Lgr5*$^+$ cells were detected in the ligaments than expected from the limiting labeling of interzone cells at E15.5, suggesting proliferation of cells in this lineage. Thus, *Lgr5*$^+$ cells in the ligament are derived from *Lgr5*$^+$ interzone cells that persisted to P21 (Figure 2F), and can be located in the synovial membrane (Figure 2F, orange arrows). Together, our findings support the notion that *Lgr5*$^+$ interzone cells are progenitor cells contributing to all structures of joints.

Col22a1, a Marker in the Articular Chondrocyte Lineage

To better understand *Lgr5*$^+$ cells, we performed transcriptome profiling using RNA sequencing (RNA-seq). We used fluorescence-activated cell sorting (FACS) to isolate three cell types: *Sox9*-GFP$^+$ cells from metacarpal cartilage

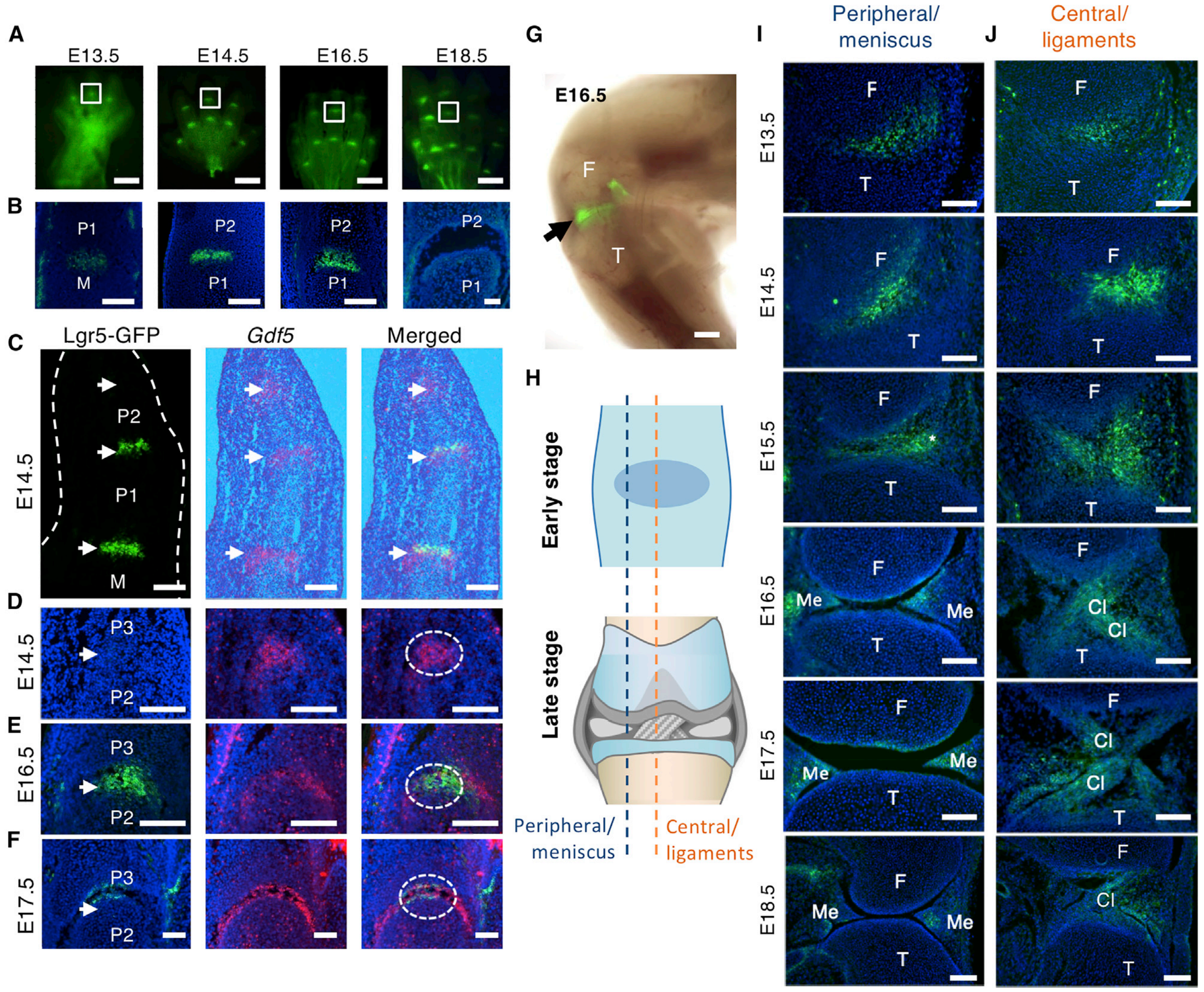

Figure 1. Expression of *Lgr5* in the Developing Digit and Knee Joints

(A) Whole-mount images of hind paws from *Lgr5-GFP* embryos (E13.5 to E18.5). Scale bars, 1 mm.

(B) Sagittal sections of the boxed areas in (A) illustrating the expression of *Lgr5* (GFP).

(C) Immunostaining for GFP (green) and *in situ* hybridization for *Gdf5* (red) of adjacent sagittal sections from digit III of E14.5 hind paw, showing *Lgr5* expression is sequential to *Gdf5* in development. *Lgr5* demarcates the center of the *Gdf5*-expressing domain in P1/P2 and M/P1 interzone.

(D–F) Higher magnifications of the P2/P3 joint showing *Gdf5* expression but not *Lgr5* at E14.5 (D), and its temporal expression in development (circled), as shown in the same joint at E16.5 (E) and E17.5 (F).

(G) Whole-mount image of the knee from an E16.5 embryo. Scale bars, 500 μm.

(H) Illustrations showing the positions and structures of the section chosen for analysis.

(I and J) *Lgr5* expression during articular cartilage/meniscus (I) and cruciate ligament (J) formation from E13.5 to E18.5.

M, metacarpal; P1, proximal phalange; P2, middle phalange; P3, distal phalange; F, femur; T, tibia; Ac, articular cartilage. Scale bars (B) to (F), (I), and (J) represent 100 μm.

anlagen of the condensed mesenchyme at E13.5 from a $Sox9^{IRES\text{-}eGFP/+}$ (Sox9-GFP) embryo, $Lgr5^{+}$ (GFP^{+}) cells from developing digit joint interzones at E14.5, and $Lgr5^{-}$ (GFP^{-}) cells from the surrounding tissues of the interzone from $Lgr5^{GFP/+}$ embryos (Figures S3A–S3C). With the cutoff set at fragments per kilobase of exon per million fragments mapped (FPKM) ≥ 5, ~8,000 genes were identified in each pool: 7,356 were common to all three datasets; some were common to two sets, and each set contained uniquely expressed genes (Figure S3E). The most commonly expressed genes specific to the $Lgr5^{+}$ bulk dataset included transcription factors (*Glis1*, *Barx2*, and *Pknox2*)

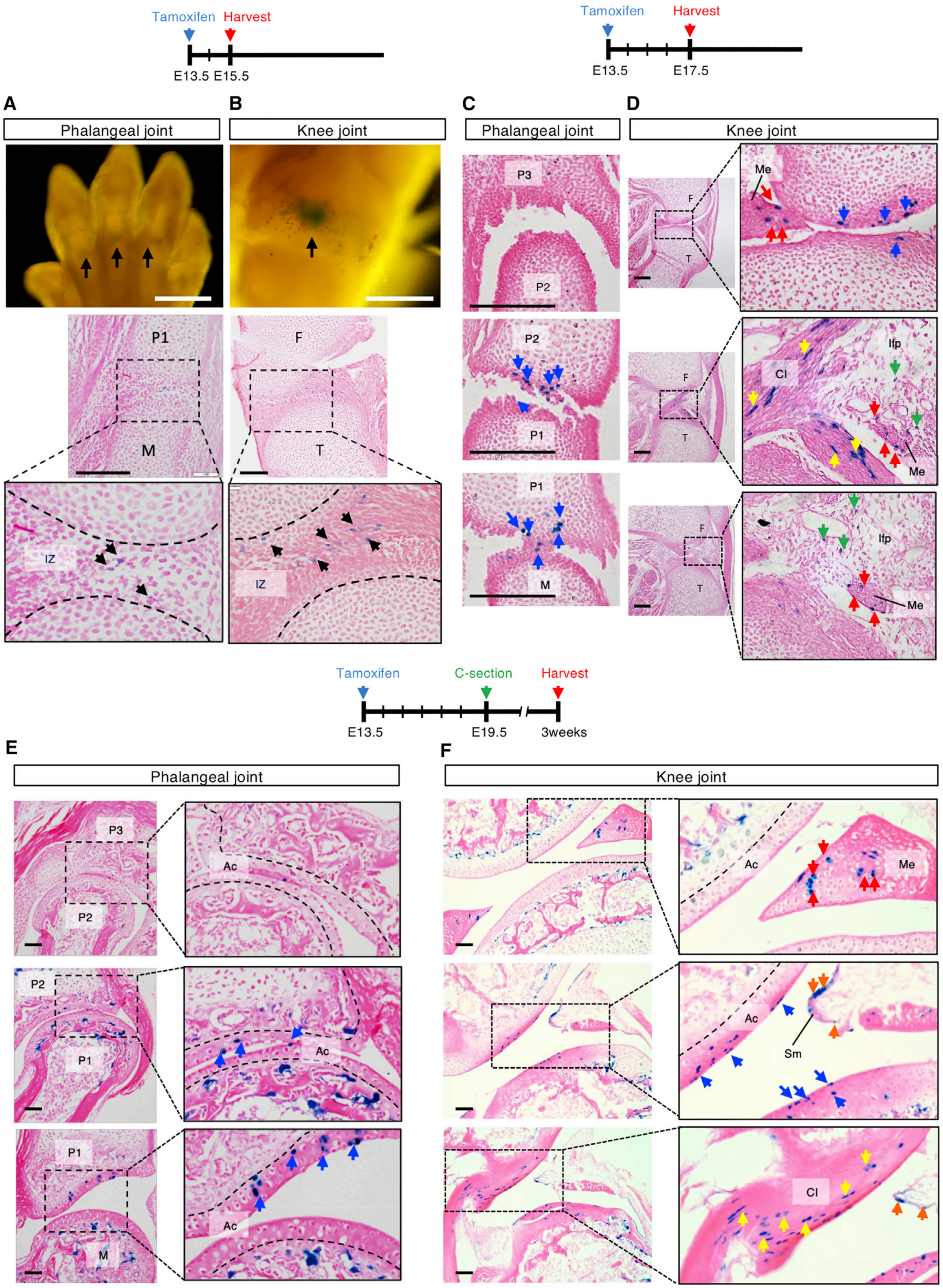

(legend on next page)

and ECM proteins (*Cilp* and *Col22a1*). Several transcription factors reported to be related to joint formation were either specifically or more strongly expressed in *Lgr5*$^+$ cells from the digit joint, such as *Gata3* (Singh et al., 2018), *Barx1/2* (Makarenkova and Meech, 2012), *Irx1/2* (Zulch et al., 2001), and *Sox5/6* (Dy et al., 2010) (Figure S3E and Tables S1–S3). Known pathways regulating interzone differentiation such as WNT, TGFβ, and MAPK (Decker et al., 2014; Gunnell et al., 2010) are also enriched in our *Lgr5*$^+$ dataset (Figure S3F and Table S4). Our data agree with descriptions of interzone cells in the literature, confirming the quality of our dataset.

Next, we looked for new information and potential markers in the lineage progression to articular chondrocytes. Analysis of ECM environment of *Lgr5*$^+$ cells can provide an indication of lineage progression with a change in the cell niche. *Cilp* and *Col22a1* are the top differentially expressed ECM genes in *Lgr5*$^+$ cells (Figure 3A), with *Col22a1* specific to *Lgr5*$^+$ cells (Figure S3E). The overall complement of ECM genes in *Lgr5*$^+$ cells indicates a chondroprogenitor phenotype not yet expressing *Comp* or *Prg4* (Figure 3A). Thus, *Cilp* and *Col22a1* are potential lineage progression markers of ECM cells. We focus on *Col22a1* (COLXXII), as it is localized to tissue junctions and detected at the superficial surface of a mature synovial joint (Koch et al., 2004). To compare the expression of *Lgr5* and *Col22a1* during joint development, we used *in situ* hybridization for *Col22a1* (Figures 3B and 3C) and immunostaining for COLXXII (Figures 3D and 3E) and GFP (*Lgr5*) proteins from E14.5 to P0 in digit and knee joints. In E14.5 P1/P2 digit joint, *Col22a1*/COLXXII expression in the *Lgr5*$^+$ interzone region was limited (Figures 3B and 3D), whereas a higher level of COLXXII was observed in the more mature M/P1 joint (Figure 3D). This indicates that the onset of *Col22a1* expression is later than *Lgr5*. Before cavitation, many cells co-express *Lgr5* and COLXXII (Figure 3D). With cavitation (E16.5), there are fewer *Lgr5*$^+$ cells and more COLXXII-expressing *Lgr5*$^-$ cells (Figure 3D, E16.5). Using the P1/P2 joint at E16.5 as an example, we clearly identified cells that express *Lgr5* but not *Col22a1* (Figure 3D, open arrowheads), double-positive cells (Figure 3D, solid arrowheads), and many cells expressing only COLXXII (Figure 3D, solid arrows). As COLXXII is an ECM protein, we define double-positive cells as cells that are GFP$^+$ (intracellular) with pericellular staining for COLXXII. This is supported by direct co-localization of *Col22a1* mRNA in *Lgr5*-expressing cells (Figure 3B). By E18.5, there were only a few *Lgr5*$^+$ cells, which are likely to be double positive for COLXXII as they are embedded in a COLXXII-enriched ECM layer (Figure S4A). At this stage, COLXXII-expressing cells marked the surface of the future articular cartilage. By birth (P0), a distinct thin layer of COLXXII-containing ECM became apparent, and the proximal/distal expression difference was no longer evident (Figure S4B). Similarly, at P10 (Figure S4C), *Lgr5*$^+$ cells were no longer detectable along the entire surface of the digit joint.

In the knee, a similar expression relationship is observed (Figures 3C and 3E). *Lgr5* is expressed at E13.5 (Figure 3E) and E14.5 (Figures 3C and 3E) as the interzone cells condense to form the articular cartilage and meniscus. *Lgr5*$^+$ cells first mark the structure of the future meniscus at E13.5, followed by the condensation of COLXXII-expressing cells along the superficial layer from E15.5 to E18.5 (Figure 3E). Differentiation to superficial meniscus cells is also likely to involve a transition from *Lgr5*- to COLXXII-expressing cells. Indeed, *Lgr5*/COLXXII double-positive cells were detected at the boundary by immunostaining at E15.5 (Figure 3E) and by *in situ* hybridization at E17.5 (Figure 3C). By E18.5 (Figures 3C and 3E), a distinct layer of COLXXII-containing matrix was present, with some *Lgr5*$^+$ cells in the deeper region of the meniscus. Similar to digit joints, *Lgr5*$^+$ cells were no longer detectable at P10, but the COLXXII layer persisted (Figure S4C). At E17.5, we detected *Lgr5*-descendant cells (LacZ$^+$) labeled on E13.5 in the COLXXII-containing superficial layer (Figure 3F) where *Col22a1* transcripts were also detected (Figure 3G), indicating that some of the cells in this layer come from *Lgr5*$^+$ interzone cells. Furthermore, cells embedded in this layer interact with the ECM as indicated by the clustering of β1-integrin into focal adhesions (Figure S4D).

Because *Lgr5* is also expressed in the developing ligaments, we analyzed its expression/localization relationship with COLXXII at E15.5, when the cruciate ligaments start to form (Figures 3H and 3I). Interestingly, while *Lgr5*$^+$ cells are detected throughout the central region of the interzone, COLXXII expression is localized to the flanking

Figure 2. Fate of *Lgr5*$^+$ Interzone Cells in Developing Synovial Joints

Pregnant mice carrying *Lgr5-GFP*;*R26R* embryos were injected with tamoxifen at E13.5, and digit and knee joints of the offspring were collected at E15.5 (A and B), E17.5 (C and D), and 3 weeks postnatal (E and F) for analysis, respectively.

(A and B) Cells in the interzone (IZ) are tagged as shown in embryos at E15.5 in whole mount (scale bars, 1 mm) and sagittal section stained for LacZ$^+$ cells (black arrows; scale bars, 100 μm).

(C–F) Descendant cells at the different stages of cell tracing at the articular surface (Ac) in both digit and knee joints (blue arrows). In the knee, LacZ$^+$ cells are also detected in the meniscus (Me; red arrows), the cruciate ligaments (Cl; yellow arrows), the infrapatellar fat pad (Ifp; green arrows), and the synovial membrane (Sm; orange arrows). Scale bars, 100 μm.

A

Gene	Fold change (Lgr5+/Lgr5-)
*Cilp**	+∞
Col22a1	+∞
Thbs4	+∞
Spon2	+∞
*Tgfbi**	+6.31
*Fmod**	+5.50
*Col8a1**	+3.25
Dcn	+3.20
Efemp1	+2.86
Col9a2	+2.21
*Ltbp4**	+2.17
Col9a3	+2.14
Col6a1	+2.02
*Ltbp3**	+1.93
Vcan	+1.81
*Wisp1**	+1.70
Col9a1	+1.64
Col11a1	+1.62
Col2a1	+1.61
*Postn**	+1.55
*Emilin1**	+1.53
*Fbln2**	+1.53
Prg4	Not expressed

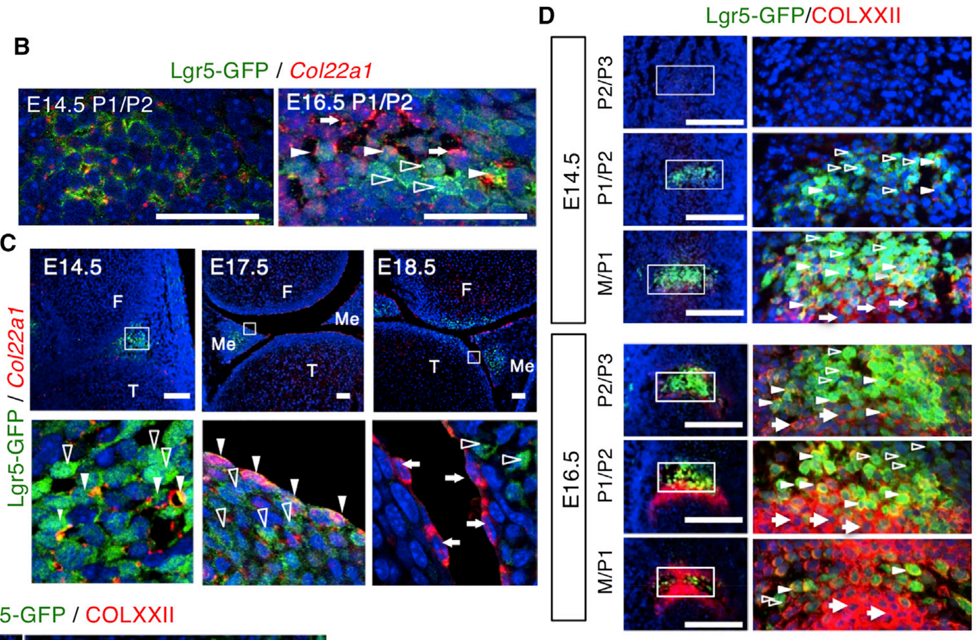

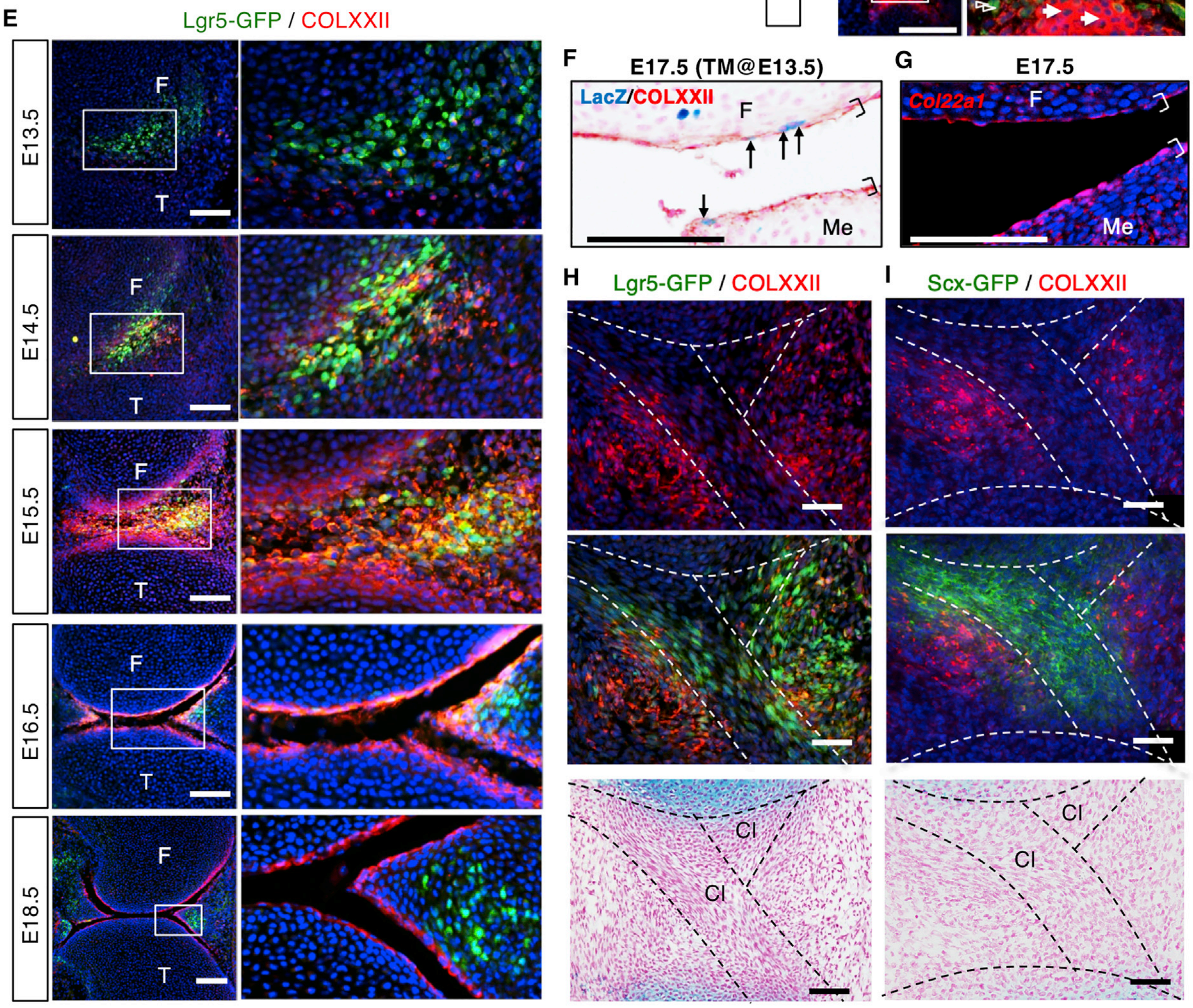

(legend on next page)

regions outside of the developing ligaments (Figure 3H). Furthermore, analysis of COLXXII expression in a *Scx*-GFP mouse showed distinct expression of *Scx* (GFP) restricted within the developing ligament and not in the flanking COLXXII-positive regions of the interzone (Figure 3I). This supports lineage divergence of *Lgr5*$^+$ progenitors to ligament cells and articular chondrocytes, with mutually exclusive expression patterns, and that there is a distinct ECM niche for these two lineages. A COLXXII-containing niche would support the formation of articular cartilage and meniscus.

Distinct Signatures of *Lgr5*$^+$ Interzone Cells for Chondrocyte or Ligament Lineages

To investigate the relationships of different cell populations in the developing knee joint, we performed single-cell RNA-seq analysis of cells from knee interzone regions of *Lgr5*$^{GFP/+}$ embryos at E14.5. After exclusion of blood cells, 5,460 interzone and surrounding cells were sequenced (Table S5). T-distributed stochastic neighbor embedding (tSNE) analysis grouped cells with similar expression profiles into six clusters. When the tSNE map was viewed in two dimensions (2D-tSNE), clusters 4 and 5 appeared to be distant from the main clusters (1, 2, 3, 6) (Figure 4A). The 3D view (3D-tSNE) showed a horseshoe shape, with clusters 1 and 5 at the tips of the horseshoe (Figure 4B). *Gdf5*, a marker for all interzone cells, is expressed in cells scattered throughout the six clusters (Figure 4C), but appeared concentrated within clusters 1, 2, 5, and 6 (Figure 4E). A total of 207 *Lgr5*-expressing cells were identified among cells expressing the endogenous *Lgr5* (n = 94), the *Lgr5-eGFP-CreER*^{T2} allele (n = 87), or both (n = 26). Interestingly, *Lgr5* expression is concentrated in clusters 1 (64% of all *Lgr5*$^+$ cells) and 5 (13%) at the tips of the horseshoe (Figure 4D), and is well illustrated in the 2D-tSNE heatmap (Figure 4F). Other *Lgr5*$^+$ cells are scattered throughout the cluster map (Figure 4D).

Next, we assessed the relationship between *Lgr5*$^+$ and *Gdf5*$^+$ cells. We expected *Lgr5*$^+$ cells to be *Gdf5*$^+$ from our *in vivo* analysis at E14.5 (Figures 1C and 1D). We observed *Gdf5*$^+$/*Lgr5*$^-$ and *Gdf5*$^+$/*Lgr5*$^+$ cells, but also *Gdf5*$^-$/*Lgr5*$^+$ cells (Figures 4G and 4H). Differentially expressed gene (DEG) analysis of *Lgr5*$^+$/*Gdf5*$^-$ and *Lgr5*$^+$/*Gdf5*$^+$ cells found the only difference was the absence or presence of *Gdf5* expression (Figure S5A). Thus, these two cell populations are the same, and the difference is likely due to a "dropout" event in single-cell RNA-seq (Kharchenko et al., 2014) for *Gdf5* if its expression was low in some *Lgr5*$^+$ cells. Similar DEG analysis of clusters 1 and 5 of *Lgr5*$^+$ cells showed that the key difference was additionally expressed genes in cluster 5 that mapped to Gene Ontology terms corresponding to cell-cycle events (Figures S5B and S5C). We selected *Ki67* as one of the most differentially expressed cell-cycle genes and mapped its expression in the 2D-tSNE profiles. A total of 14% of the *Lgr5*$^+$ cells in all clusters expressed *Ki67*; most of them are in cluster 5 but not in cluster 1 (Figure 4I). Similarly, we found that 12.2% ± 2.1% (n = 3) of *Lgr5*$^+$ cells in the E14.5 knee interzone region expressed Ki67; these cells were located around the periphery of the *Lgr5*$^+$ interzone region (Figure 4J).

As most *Lgr5*$^+$ cells were in cluster 1, we conducted a DEG analysis of cluster 1 against the other five clusters. We identified 110 DEGs (Figure 4K and Table S6), including transcription factors related to joint development such as *Osr2*, *Trps1*, *Barx1*, and *Sox4*. When we increased the stringency by comparing *Lgr5*$^+$ cells in cluster 1 with *Lgr5*$^-$ cells

Figure 3. Dynamic Spatiotemporal Expression of Lgr5 and Collagen XXII in Knee Joint Development

(A) Matrix genes with higher expression in *Lgr5*$^+$ compared with *Lgr5*-from digit bulk transcriptome. "+∞" indicates that a gene is not expressed in the *Lgr5*$^-$ cell population. Genes with asterisks indicate that expression data for the developing joint are available in Eurexpress.

(B and C) Co-expression of *Lgr5* (GFP) and *Col22a1* mRNA in digit (B; scale bars, 50 μm) and knee joints (C; scale bars, 100 μm); boxed areas are shown in the bottom panels. Cells positive to Lgr5-GFP only are indicated with open white arrowheads, double positive to Lgr5-GFP/*Col22a1* with solid white arrowheads, and cells expressing only *Col22a1* with solid arrows.

(D) Double fluorescent immunostaining of *Lgr5-GFP* (green) and COLXXII (red) on sagittal sections of the developing knees of *Lgr5-GFP* embryos at E14.5 and E18.5 (see also Figures S4A and S4B). Images of the boxed areas are shown on the right panels. Formation of the articular cartilage progresses from Lgr5$^+$ cells (open white arrowheads), to cells expressing both Lgr5 and COLXXII (solid white arrowheads), to cells expressing only COLXXII (solid arrows). Scale bars, 200 μm.

(E) Co-localization of *Lgr5* and COLXXII in developing articular cartilage and meniscus. COLXXII matrix layer condenses to a very narrow region at the superficial surface of the fully developed articular cartilage and meniscus. Boxed regions are shown in the right panels. Scale bars, 200 μm.

(F) LacZ$^+$ descendant cells of Lgr5 (arrows), with tamoxifen (TM) injected at E13.5 and harvested at E17.5 can be detected at the COLXXII$^+$ superficial layer (brackets) of the articular cartilage and meniscus. Scale bars, 100 μm.

(G–I) All cells in this layer (G; brackets) express *Col22a1* in a comparable section. Co-localization of COLXXII with Lgr5 (GFP) (H) and *Scx* (GFP) (I). Histology of the same sections stained with Alcian blue and nuclear fast red (H and I, lower panels). F, femur; T, tibia; Cl, cruciate ligament. Scale bars, 50 μm.

See also Figures S3 and S4.

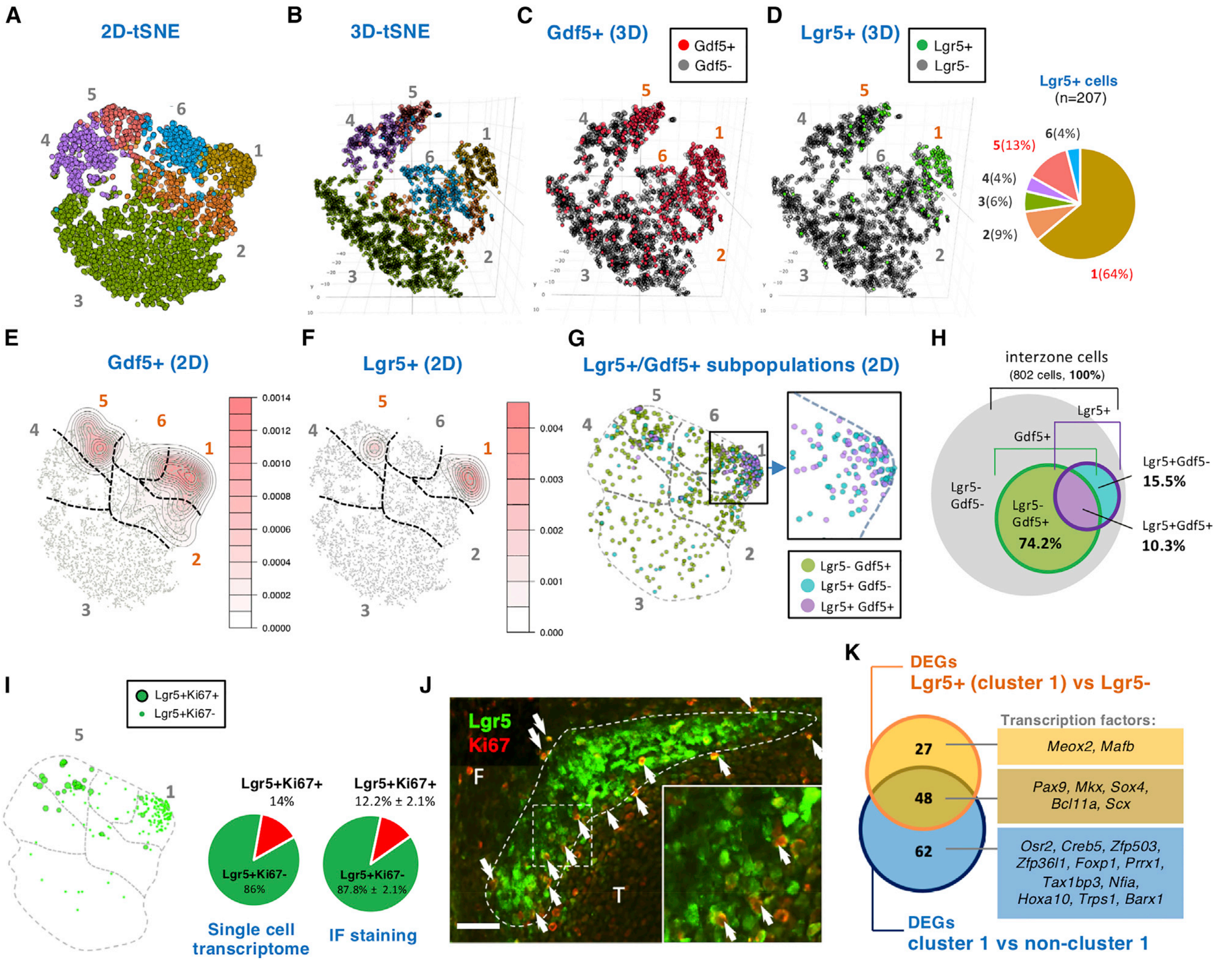

Figure 4. Properties and Molecular Signatures of *Lgr5*$^+$ Cells in E14.5 Knee Joint Interzone Revealed by Single-Cell Transcriptome

(A and B) Gene expression from 5,460 cells was analyzed using tSNE plots in 2D (A) or 3D (B), with six major clusters identified.

(C and D) 3D view for the distribution of *Gdf5*$^+$ (C) and *Lgr5*$^+$ (D) cells. The relative distribution of *Lgr5* expression in the different clusters is presented as a pie chart in (D).

(E and F) Contour plots illustrating the density of cells expressing (F) *Lgr5* shown in (B), and (E) *Gdf5* shown in (C), with the corresponding colored density chart.

(G) 2D-tSNE showing the distribution of *Lgr5*$^+$ among *Gdf5*$^+$ cells. Higher magnification of the boxed area in cluster 1 is shown on the right panel, highlighting the relative *Lgr5*$^+$/*Gdf5*$^-$ (cyan) and *Lgr5*$^+$/*Gdf5*$^+$ (purple) cells.

(H) Venn diagram illustrating the distribution and percentages of *Lgr5*$^-$/*Gdf5*$^+$ (green), *Lgr5*$^+$/*Gdf5*$^-$ (cyan), and *Lgr5*$^+$/*Gdf5*$^+$ (purple) subpopulation of cells.

(I) 2D-tSNE plot highlighting the distribution of *Lgr5*$^+$/*Ki67*$^+$ in cluster 5, relative to the dominance of *Lgr5*$^+$/*Ki67*$^-$ cells in cluster 1, and the percentage of *Lgr5*$^+$/*Ki67*$^+$ shown in a pie chart.

(J) Double immunofluorescence detection of Lgr5-GFP- and Ki67-expressing cells in an E14.5 knee joint. The peripheral of the Lgr5 cell population is marked with a dotted line, and arrows indicate the double-positive cells with the boxed region shown in higher magnification. The relative percentage of Lgr5$^+$/Ki67$^+$, determined from three embryos, is also shown in a pie chart. F, femur; T, tibia. Scale bars, 20 μm.

(K) Venn diagram showing the DEGs of cluster 1 versus non-cluster 1 (n = 110, blue circle) and DEGs *Lgr5*$^+$ versus *Lgr5*$^-$ cells (n = 75, yellow circle). There are 48 common DEGs identified with these two gene lists. Transcription factors in each sector are shown.

See also Figure S5 and Table S6.

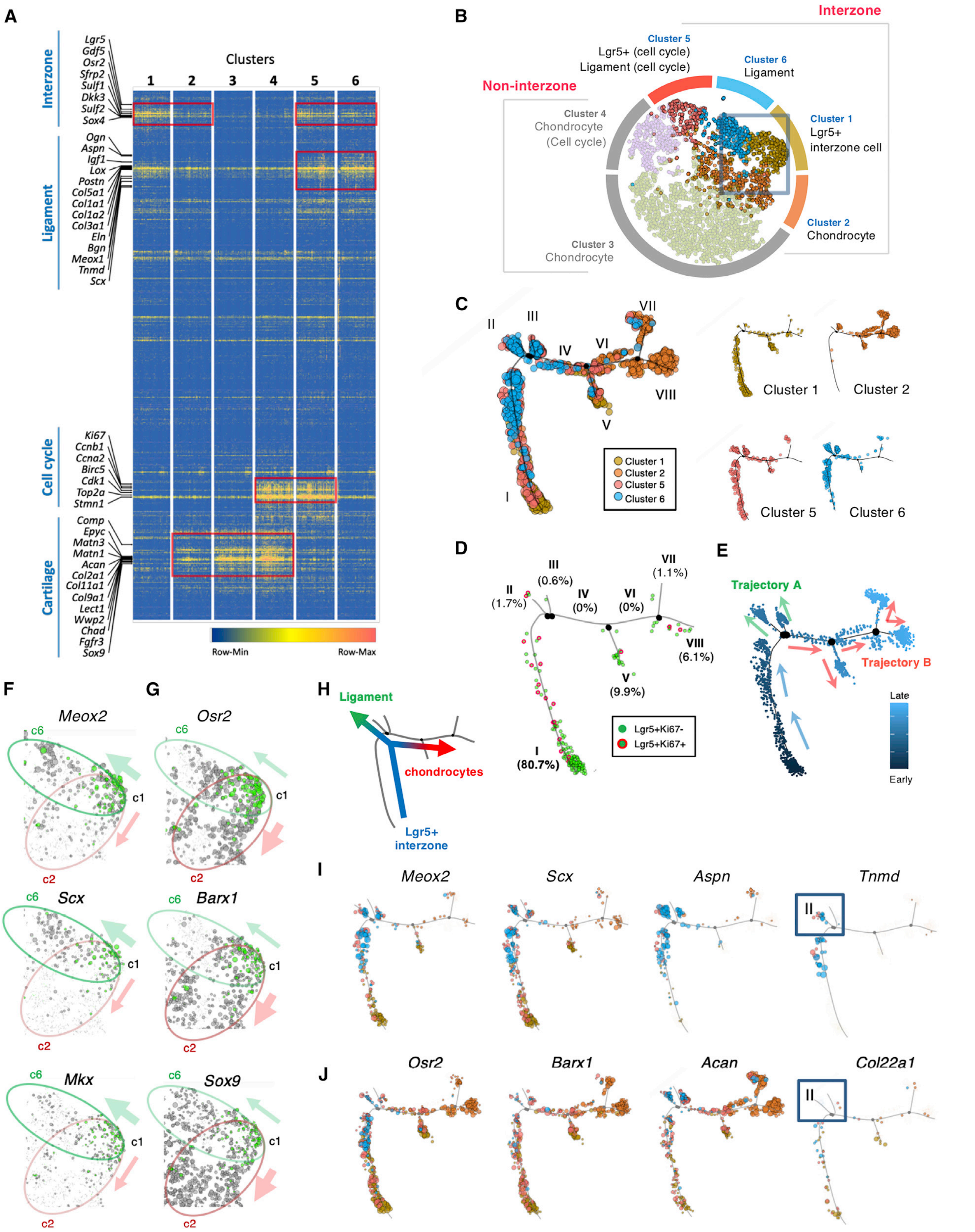

(legend on next page)

in the whole population, we identified 75 DEGs (Table S6), 48 of which overlapped with the previous gene set including the transcription factors *Mkx* and *Scx*. Twenty-seven genes were specifically expressed in *Lgr5*$^+$ cells, including the transcription factor *Meox2* (Figure 4K). Together, these analyses described distinct signatures of *Lgr5*$^+$ cells in the interzone for the chondrocyte and ligament lineages.

Trajectories of Joint Lineage Specification

To annotate these clusters, we clustered the top 5% of the dispersed genes (genome-wide of all 5,460 cells) into a heatmap. Gene modules associated with interzone, ligament, cell cycle, and cartilage were identified (Figure 5A). As expected from the 2D-tSNE heatmap for *Gdf5* expression (Figure 4E), cells in clusters 1, 2, 5, and 6 expressed genes associated with interzone cells, including *Gdf5*, *Osr2*, *Sfrp2*, *Sulf1*, and *Sox4*. Relative to other clusters, cells in clusters 5 and 6 expressed higher levels of genes related to ligament, such as *Lox*, *Col1a1*, *Aspn*, and *Scx*. The major difference between cluster 5 and cluster 6 was the high abundance of cell-cycle-related genes in cluster 5, such as *Ki67*, *Ccna2*, *Birc5*, and *Top2a*, which are also expressed in cluster 4. Cells in clusters 2, 3, and 4 expressed cartilage-related genes such as *Epyc*, *Matn1*, *Acan*, *Lect1*, and *Sox9*. When we analyzed the same dataset with principal component analysis, we found similar clustering of interzone and non-interzone cells as well as similar cell-cycle status of cells in these clusters (Figures S5D–S5G). The expression profile in each of the clusters agreed with the violin plots of representative genes for cartilage and ligament, and cell-cycle genes (Figure S5H). Thus, clusters 1, 2, 5, and 6 are considered "interzone clusters," whereas 3 and 4 are defined as "non-interzone clusters" (Figure 5B). Cluster 1 is enriched with *Lgr5*$^+$/*Gdf5*$^+$ interzone cells and is mapped between the clusters with the ligament (cluster 6) and chondrocyte (cluster 2) signatures, with some "mixing" of cells at the borders, implicating a potential divergence of the cluster 1 interzone cells into either chondrocytes or ligament lineages (Figure 5B).

To study the cell-lineage divergence, we performed a pseudo-timeline analysis focusing on the interzone clusters (1, 2, 5, and 6) that mapped into eight potential cell states (Figure 5C). Cells in cluster 1 mapped mainly in state I with some cells scattered throughout other states. Cells in clusters 5 and 6 with the ligament signature were distributed in states I, II, and III, while cells in cluster 2 with the chondrocyte signature were distributed primarily in cell states V, VI, VII, and VIII (Figure 5C). State IV contained a mixture of cells from all four clusters. Next, we assessed the distribution of *Lgr5*$^+$/*Ki76*$^+$ cells and showed that they are distributed within *Lgr5*$^+$/*Ki67*$^-$ cells, but most *Lgr5*$^+$ cells mapped to state I (Figure 5D), which represents the most primitive/young state as predicted in the pseudo-timeline (Figure 5E). Two major trajectories branched from state I: trajectory A consisted of cells in states II/III (Figure 5E, green arrows) and trajectory B included cells in states V/VI/VII/VIII (Figure 5E, red arrows).

To characterize the trajectories, we assessed expression of the differentially expressed transcription factors identified (Figure 4K). *Scx*, *Meox2*, and *Mkx* are expressed in ligament cells, and their expression is enriched in cells of clusters 1 and 6 (Figure 5F, green oval) but less in cells of cluster 2 (Figure 5F, pink oval). *Osr2* and *Barx1* are expressed in cartilage development and their expression is enriched in clusters 1 and 2, but less in cluster 6 (Figure 5G). *Sox9*, a key chondrogenic transcription factor, is not differentially expressed in cluster 1 or among *Lgr5*$^+$ cells: overall, its expression is up-regulated in cluster 2 but not in cluster 6 (Figure 5G). This supports a divergence of cells in cluster 1 from a "bipotential" state that can differentiate along the ligament or the articular cartilage lineage (Figure 5H). Next, we mapped the transcription factors and ECM genes specific to ligament (Figure 5I) and cartilage (Figure 5J) onto the pseudo-timeline and found them to be enriched in the cell states that supported the relevant tissue trajectory (Figure 5E). Based on the algorithm for the trajectory, some

Figure 5. Lineage Trajectories of Knee Joint

(A) Heatmap showing the clustering of the top 5% dispersed genes (genome-wide), from the profiles of 200 cells randomly selected from each cluster as a representative. Gene clusters related to joint development, ligament, cell cycle, and cartilage were identified.

(B) Summary diagram of the property/signature of each cluster, with clusters 1, 2, 5, and 6 defined as interzone cells.

(C) Pseudo-timeline analysis of cells from clusters 1, 2, 5, and 6 identified eight cell states, and the distribution of cells from individual cluster and a combined map along the pseudo-timeline is shown.

(D) Distribution of all *Lgr5*$^+$ cells along the pseudo-timeline, and relative percentages distributed in each of the cell state indicated, together with proliferative *Lgr5*$^+$/*Ki67*$^+$ cells.

(E–G) The pseudo-timeline (E) predicted a gradient of "early" (dark blue) to late (light blue) cells, with a major divergence to two trajectories A and B, with arrows indicating the predicted direction. Expression distribution of ligament (F) and chondrocyte (G) genes are mapped onto the 2D tSNE. The size of each circle is a relative reflection of the gene expression level; *Lgr5*$^+$ cells are indicated as green.

(H) Illustration showing the major lineage divergence of *Lgr5*$^+$ interzone cells.

(I and J) Distribution of cells expressing ligament (I) and cartilage (J) genes along the pseudo-timeline. The boxes highlight a specific comparison of cells in state II expressing a mature ligament marker *Tnmd* but not *Col22a1*.

cells co-expressed both ligament and cartilage transcription factors prior to the lineage divergence. For example, cells from cluster 6 distributed at the end of state I expressed both *Meox2* (blue circles in Figure 5I, left panel) and *Osr2* (blue circles in Figure 5J, left panel). In support of the *in vivo* data (Figure 3H), state II cells in trajectory A did not express *Col22a1* but did express the mature ligament marker *Tnmd* (Figures 5I and 5J, blue boxes). *Col22a1* was expressed in cells of trajectory B, supporting our hypothesis that *Col22a1* marks the chondrocyte lineage as distinct from the ligament lineage. Thus, our single-cell transcriptome data, together with our lineage-tracing experiment, showed $Lgr5^+$ interzone cells are likely to be multipotent and represent a stage of joint formation at which the lineage divides into ligament and articular chondrocytes (Figure 5H).

$Lgr5^+$ Interzone Tissue Repairs Cartilage Lesion

$Lgr5^+$ interzone cells could be suitable for the repair of cartilage, as they are "primitive" and multipotent. To test this capacity, we dissected the $Lgr5\text{-}GFP^+$ interzone tissue from E13.5 *Lgr5-GFP;ROSA-tdTomato* embryos and transplanted it to a full-thickness needle-puncture lesion at the trochlear groove of the knee of 8-week-old mice (n = 3) (Figures 6A and S6B). Fifteen days post puncture, $tdTomato^+$ cells were detected in the lesion, as differentiating round chondrocytes in the intermediate and deep zones of articular cartilage (Figures 6B and 6C, arrows; control section is shown in Figure S6A) and flattened cells in the superficial zone (Figures 6B and 6C, arrowheads). The healing lesion contained cells that originated from the transplanted tissue, which produced proteoglycan-enriched matrix (Figure 6C) and COL II deposition (Figure 6D). The new tissue integrated well with the host cartilage (Figure 6C, box) with a new superficial layer containing COLXXII and CILP1 intercalated with the superficial zone of the host articular cartilage (Figures 6E and 6F). The implanted tissue suppressed expression of COL I (Figure 6G) in the lesion, which could result in unwanted fibrosis during cartilage repair. Together, our findings suggest that $Lgr5^+$ interzone cells have repair capacity for articular cartilage.

DISCUSSION

The diverse tissues of the synovial joint derive from mesenchymal cells in the developing interzone, most or all of which express *Gdf5* (Koyama et al., 2008). Tracing studies in a $Gdf5\text{-}CreER^{T2}$ mouse showed that after the initial dedifferentiation of chondrogenic cells to $Gdf5^+$ interzone cells, further expansion of the $Gdf5^+$ region is primarily through recruitment of regional mesenchymal cells rather than proliferation of $Gdf5^+$ interzone cells (Shwartz et al., 2016). Furthermore, Shwartz et al. (2016) proposed that cells within the interzone have contextual cues, and their ability to produce distinct joint tissues is governed by positional effects. Here, we identified a new subset of *Gdf5*-expressing interzone cells, distinguished by co-expression of *Lgr5*, a known marker for highly proliferative stem cells (Leung et al., 2018), although cell division is rare within the interzone (Shwartz et al., 2016). Significantly, this subset of *Lgr5*-expressing cells in the $Gdf5^+$ population can also give rise to the diverse tissues of the joint, including the synovial membrane, cruciate ligaments, meniscus, and articular cartilage.

$Lgr5^+$ Interzone Cells Contribute to the Making of the Diverse Joint Structures

The $Lgr5^+$ cell pool emerges shortly after the initial appearance of *Gdf5*-expressing cells marking the future joint site. Therefore, $Lgr5^+$ cells are likely to be derived from $Gdf5^+$ cells that have undergone dedifferentiation, consistent with cell-fate mapping findings using *Gdf5*-Cre mice (Koyama et al., 2008), and that the progressive differentiation from a $Gdf5^+$ to a $Lgr5^+$ cell is a sequential event. In the digit joints, because $Lgr5^+$ cells arise and remain localized within the center of the interzone, incoming $Gdf5^+$ cells proposed in the continuous influx model (Shwartz et al., 2016) would be the peripheral to the central pool of $Lgr5^+$ cells. Our single-cell transcriptome showed that the $Lgr5^+$ pool expands in part through differentiation and proliferation of the peripheral interzone cells, shown by the presence of $Lgr5^+/Ki67^+$ cells in the developing joint, and a population of $Lgr5^+$ cells that differ only in the expression of active cell-cycle-related genes in the tSNE map of cell clusters. Other mechanisms might exist in the more complex knee interzone.

In our mapping study, activation of the *LacZ* at E13.5 would tag this early pool of $Lgr5^+$ cells, and we observed that they can contribute to all structures of the joint. Having only a few *Lgr5*-expressing cells tagged from a single tamoxifen injection allows us to assess the level of contribution to the different structures of the joint. In the developing knee joint, there were no observable differences in the contribution of $Lgr5^+$ cells to the articular cartilage, meniscus, and synovium. Interestingly, many more descendant cells are found in the cruciate ligaments, suggesting that they have proliferated. Furthermore, *Lgr5* continues to be expressed in cells of the developing ligament, consistent with expansion of the tagged $Lgr5^+$ cells from the E13.5 interzone with numerous descendant cells in the ligament at E17.5. This is supported by the identification of a ligament cell cluster with active cell-cycle genes in the tSNE map of the single-cell transcriptome data from an E14.5 interzone.

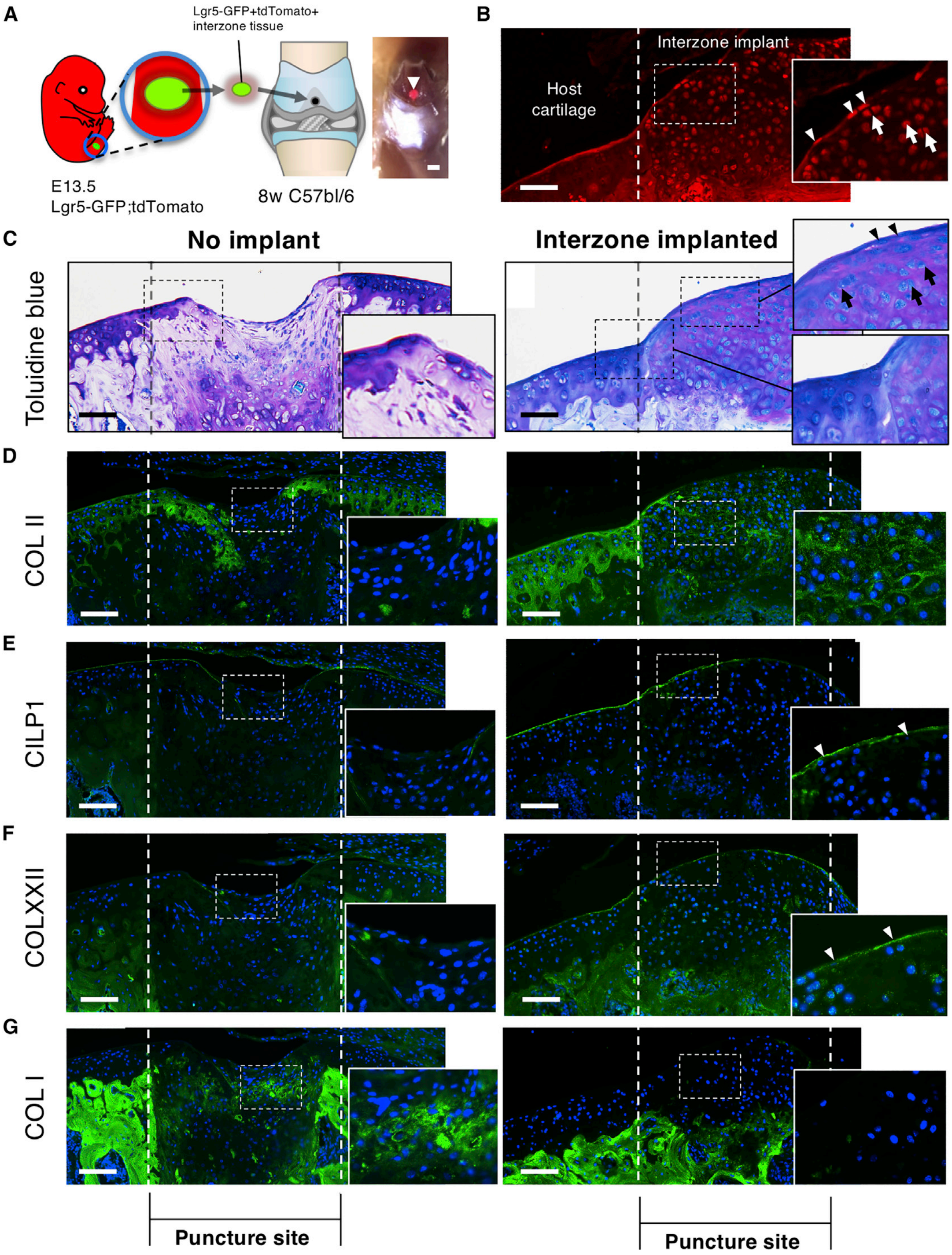

Figure 6. Capability of *Lgr5*$^+$ Interzone Tissue to Repair Cartilage Lesion

(A) Experimental design for the isolation of *Lgr5*$^+$ interzone tissue (labeled with both GFP and tdTomato) and transplantation into articular cartilage defect for repair. The right panel shows the tdTomato$^+$ tissue inside the lesion directly after transplantation. Scale bars, 1 mm.

(B) Implanted interzone tissue (tdTomato$^+$) integrated with the host cartilage and differentiated into cells in articular cartilage and flattened cells in the superficial layer (arrowheads), 15 days post implantation.

(C–G) Histological (C) and ECM marker (D–G) analyses performed on lesion with and without *Lgr5*$^+$ implantation after 15 days. High magnifications of the boxed areas are shown in the insets.

Scale bars, 50 μm. See also Figure S6.

Lgr5$^+$ Cells Are Poised Progenitors at the Onset of Lineage Divergence

Consistent with the *in vivo* analyses, the transcriptomic data from both the bulk and single-cell analyses support *Lgr5*$^+$ cells as progenitors "poised" for differentiation along the articular chondrocyte or ligament lineages. Using the clusters representing the interzone cells to derive a pseudo-timeline, we identified eight cell states, placing cluster 1 with the bulk of the *Lgr5*$^+$ cells as the most "primitive" state I of "poised" progenitor cells (Figure 5D). Many *Lgr5*$^+$ cells in state I express gene signatures for both ligament and chondrocyte, then specialize into the more distinct signature for the ligament or chondrocyte lineages. Changes in the balance of lineage-specifying transcription factors may control the divergence.

Interestingly, about 10% of *Lgr5*$^+$ cells are found in state V, predicted to be derived from state I, and the reason for this is unclear. It is possible that these are from cells recruited differently in joint development (Shwartz et al., 2016), and this cannot be excluded. Multiple cell states exist within each of the ligament or chondrocyte trajectories, suggesting that there are subclasses of cells within each lineage, possibly related to their position along the cruciate ligament, or differences between chondrocytes residing in the articular or meniscus regions. For example, a multipotent *Scx*$^+$/*Sox9*$^+$ progenitor cell pool at the chondro-tendinous/ligamentous junction gives rise to tenocytes/ligamentocytes and chondrocytes (Sugimoto et al., 2013). We propose that there could be positional values for these cells within the developing knee joint, as depicted in a model based on our current findings (Figure 7).

Role of Lgr5 and Wnt Signaling in Joint Development

Although Lgr5-GFP homozygous mice are perinatal lethal (Barker et al., 2007), they show no histological abnormality in joints (Figure S2). This might be due to functional redundancy among the three LGR proteins (*LGR4, LGR5*, and *LGR6*) (Ruffner et al., 2012). *Lgr4* is expressed in our transcriptome dataset, although *Lgr6* is not. *Lgr5* expression may reflect a positional effect at an appropriate level of Wnt ligands for its activation, and the potentiation of Wnt signaling by *Lgr5* in turn sustains a higher signaling level through interaction with R-spondins (de Lau et al., 2011), necessary for the progression to the next stage, poised for the formation of other structures and cavitation.

Col22a1 Marks an Articular Cartilage Lineage from Lgr5-Expressing Interzone Cells

Decreasing *Lgr5* expression with cavitation and in cells at the juvenile articular surface is consistent with the need to reduce anti-chondrogenic Wnt signal for articular chondrocyte differentiation. We know very little about the molecular control of this lineage. Chondrogenic bone morphogenetic protein signaling plays an important role (Rountree et al., 2004), supported by the continuing expression of *Gdf5* while *Lgr5* decreases, tipping the balance to chondrogenesis, and our identification of *Col22a1* as an intermediate marker for this lineage and cells co-expressing *Col22a1* and *Lgr5*. *Col22a1*/COLXXII expression started proximally, but then occurred close to the margins of the future articular surface within the developing interzone. This is best illustrated in the less complex digit joints, and is consistent with the proposed model whereby the developing interzone is organized into three layers, with the two outer layers containing cells committed to become articular chondrocytes, and cells in the middle layer contributing to other structures or undergoing apoptosis (Mitrovic, 1977). The COLXXII-expressing cell layers within the interzone are broad just prior to cavitation. Overlap between the COLXXII-expressing regions and *Lgr5*$^+$ cells at the earliest stage of cavitation suggests that most *Col22a1*-expressing cells are derived from *Lgr5*$^+$ cells. Interestingly, the *Lgr5*$^+$/*Col22a1*$^+$ co-expressing cells are primarily localized to the mid region of the articular surface throughout the cavitation process, with *Col22a1*$^+$/*Lgr5*$^-$ cells occupying the flanking regions along the entire surface of the joint. If *Col22a1*-expressing cells are derived from *Lgr5*$^+$ cells, expansion of the articular surface is mediated through cellular differentiation at the midpoint contributing to the lateral growth of the joint surface. Indeed, this may have been reflected in the multiple cell states identified in the chondrocyte trajectory from the pseudo-timeline prediction of the single-cell transcriptome (Figures 5D and 5E).

We propose a model whereby cells express *Gdf5*, progress to *Gdf5*$^+$/*Lgr5*$^+$ double-positive progenitor cells, then *Lgr5*$^+$/*Col22a1*$^+$ double-positive committed articular chondrocyte progenitors, and finally *Col22a1*-expressing juvenile articular chondrocytes (Figure 7). The continuous influx model invokes an expansion of the *Gdf5*$^+$ interzone cell pool from recruitment (Shwartz et al., 2016). We propose that a spatial effect of the various pools of committed progenitors dictates the positioning of the joint structures. Single-cell transcriptome with more cells from additional developmental time points should provide key markers for validation, and further insights into the molecular controls and subdivision of cell states.

Capacity of *Lgr5*$^+$ Cells in Cartilage Repair: Re-establishment of the Collagen XXII-Containing Superficial Layer

The superficial zone contains cells producing Lubricin/*Prg4* for joint lubrication (Kozhemyakina et al., 2015) and progenitor cells (Dowthwaite et al., 2004). Recent cell-tracing studies identified self-renewing progenitor cells at the superficial zone of the mouse articular cartilage that may undergo symmetric and asymmetric expansion in juvenile

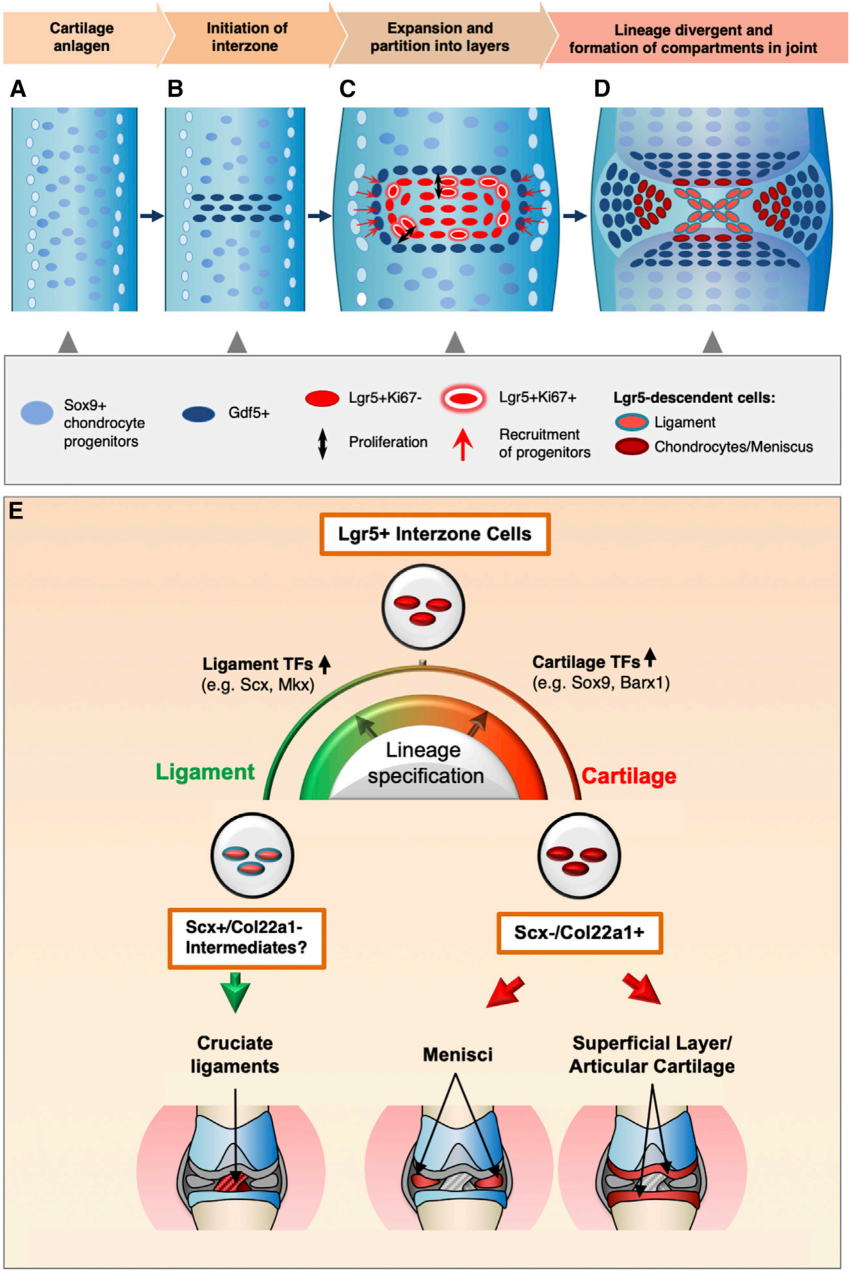

Figure 7. Model for the Contribution and Divergence of Progenitor Cells in Joint Formation

A schematic diagram showing the developmental processes of a knee joint.

(A) Formation of the cartilage anlagen containing *Sox9*-expressing chondrocytes.

(B) Dedifferentiation of *Sox9*$^{+}$ chondrocytes to *Gdf5*$^{+}$ interzone cells at the presumptive joint site.

(C) Expansion of the interzone through recruitment of mesenchymal cells from the surrounding and limited proliferation of peripheral *Lgr5*$^{+}$ cells, and the partitioning of the interzone into specific regions.

(D) *Lgr5*$^{+}$ interzone cells undergo lineage divergent and differentiate into cells for the different tissues in a mature joint.

(E) *Lgr5*$^{+}$ interzone cells are multipotent progenitors contributing to all interior structures of the knee joint including cruciate ligaments, articular cartilage, and menisci. *Lgr5*$^{+}$/*Scx*$^{-}$/*Col22a1*$^{+}$ cells are committed progenitors for articular chondrocyte lineage and menisci, whereas *Lgr5*$^{+}$/*Scx*$^{+}$/*Col22a1*$^{-}$ cells are restricted within the ligament lineage.

joints (Decker et al., 2017; Li et al., 2017). Our analysis showed that COLXXII demarcates the outermost surface of the joint. Cells embedded in the COLXXII-containing layer have a distinct flattened morphology with direct interaction with the ECM, shown by the presence of focal adhesions with clustering of β1-integrin receptors. These cells might provide a special niche for cell maintenance and act as a source of progenitors. Finally, we showed that *Lgr5*$^{+}$ cells collected from an E13.5 embryonic knee joint can repair a full-thickness articular cartilage defect. Importantly, a COLXXII-containing superficial layer was re-established in the healing defect, which quickly integrated with the host cartilage, indicating that these *Lgr5*$^{+}$ cells are candidates for cell-based therapy for cartilage and ligament trauma or associated degenerative diseases.

EXPERIMENTAL PROCEDURES

Mouse Strains

Lgr5-eGFP-IRES-CreER^{T2} (Lgr5-GFP) (Barker et al., 2007), *ROSA26-LacZ* (R26R) reporter (Soriano, 1999), and *Scx-GFP* (Pryce et al., 2007) mice were maintained in C57bl/6 background. PCR primers

for genotyping these mice are listed in Table S7. All animal works were approved by the Committee on the Use of Live Animals in Teaching and Research of the University of Hong Kong.

Real-Time qPCR

mRNA was extracted from E14.5 digit interzone and surrounding non-interzone regions using TRIzol reagent (Thermo Fisher). cDNA was generated using the PrimeScript RT reagent kit (Clontech). qPCR was performed using LightCycler480 SYBR Green I Master kit (Roche). All primers are listed in Table S7.

In Situ Hybridization and Immunohistochemistry

In situ hybridization was performed as previously described (Gao et al., 2009), using [α-^{35}S]uridine triphosphate-labeled riboprobes for *Gdf5*. Fluorescence *in situ* hybridization was performed as previously described (Shwartz and Zelzer, 2014), using a digoxigenin-labeled probe for *Col22a1*. For immunohistochemistry, sections were incubated with primary antibodies for goat anti-GFP (ab6673), guinea pig anti-COLXXII (produced by Dr. Manuel Koch), sheep anti-CILP1 (R&D Systems), and rabbit anti-Ki67 (Abcam ab1558), and detected using the relevant secondary antibodies (Alexa Fluor 488 anti-rabbit [Thermo Fisher], Alexa Fluor 488 anti-goat [Abcam], Alexa Fluor 488 anti-sheep [Abcam], and Cy3 anti-guinea pig immunoglobulin G [Jackson ImmunoResearch]).

Lineage Tracing and X-gal Staining

Tamoxifen (Sigma) was administered by intraperitoneal injection (0.2 mg/g body weight) to pregnant mice carrying Lgr5-GFP/R26R embryos at E13.5. Limbs were processed for whole-mount X-gal staining to detect LacZ activity. Three-week-old samples were decalcified overnight in 0.5 M EDTA (pH 7.5); 7-μm paraffin sections were counterstained with eosin.

Imaging

Images were acquired using an Axioplan 2 microscope (Zeiss) with SPOT-camera or the Leica TCS SPE Live confocal microscope. The contrast and color of some images were adjusted with the "brightness/contrast" and "hue/saturation" functions in Adobe Photoshop CS. No further modifications of the images were made.

Bulk Transcriptome Analysis

Sox9^{+} cells were isolated from digits of E13.5 *Sox9-GFP* embryos (Nakamura et al., 2011). *Lgr5*$^{+}$ and surrounding non-*Lgr5* (*Lgr5*$^{−}$) cells were isolated from forelimb digit interzones of E14.5 *Lgr5*$^{GFP/+}$ mice (Figures S3A–S3C). Cells were released with a mixture of TrypLE Express (Gibco) and 0.1% DNase I for 20 min, filtered through a 40 μm cell strainer, and sorted by FACS using an Aria I flow cytometer (BD Biosciences). Total RNA was extracted using a mirVana miRNA Isolation Kit (Ambion) and cDNA libraries were constructed with 10 ng of RNA using a SMARTer Ultra Low Input Kit (v3, Illumina), and sequenced using the Illumina HiSeq 1500 platform (Center for Genomic Sciences, The University of Hong Kong). cDNA fragment sequences were aligned to mouse genome (mm10) using the HISAT program (Kim et al., 2015). FPKM values were generated for comparison (Figure S3D). Genes with FPKM $\geq$ 5 were considered as expressing genes. Pathway and expression analyses were performed with DAVID (Huang da et al., 2009) and Eurexpress (Diez-Roux et al., 2011) databases, respectively. Datasets have been uploaded to the Gene Expression Omnibus for public access (GEO: GSE110281).

Single-Cell Transcriptome Analysis

The knee interzone and surrounding cartilage tissues were dissected with the aid of GFP expression from E14.5 *Lgr5*$^{GFP/+}$ embryos. Dissociated cells were pooled for single-cell RNA sequencing with 10X Genomics Chromium Single Cell Controller for encapsulations (10X Genomics). cDNA libraries were prepared according to the manufacturer's instructions (Chromium Single Cell 3′ Reagent Kits v2 and Chromium Single Cell A Chip Kit), then sequenced on the Illumina HiSeq 1500 platform. The raw data were processed with the Cell-Ranger pipeline (version 2.1.0; 10X Genomics) for alignment to mm10, quantification of UMIs (unique molecular identifiers), and dimension-reduction (tSNE) analysis. Data were aligned to CreER^{T2} sequence to assess the expression of the *Lgr5-eGFP-CreER*^{T2} allele. Cells expressing *Lgr5* and/or *Lgr5-eGFP-CreER*^{T2} alleles are considered as *Lgr5*$^{+}$ cells. Clustering and pseudo-timeline analyses were performed with Cell-Ranger and Monocle2, respectively. Details of the parameter settings are shown in Table S5.

Cartilage Puncture and Repair with Lgr5^{+} Interzone Tissue

Lgr5-GFP mice were crossed with *ROSA26-tdTomato* mice to generate double heterozygous *Lgr5-GFP*;*tdTomato* embryos. GFP^{+} tissue was dissected from the forming knee joint of E13.5 embryos and transplanted to a lesion punctured with a 27-gauge needle at the trochlear groove of 8-week-old C57bl/6 mice (n = 3, Figure 6A). Punctured animals without tissue transplant represented the control.

ACCESSION NUMBERS

The accession number for the bulk transcriptome datasets reported in this paper is GEO: GSE110281. The accession number for the single-cell transcriptome dataset reported in this paper is GEO: GSE130919.

SUPPLEMENTAL INFORMATION

Supplemental Information can be found online at https://doi.org/10.1016/j.stemcr.2019.08.006.

AUTHOR CONTRIBUTIONS

C.F., W.C.W.C., Y.L. and D.C. designed the experiments, analyzed the data, and wrote the manuscript. C.F., W.C.W.C., and Y.L. performed *in vivo* experiments. W.C.W.C. was involved in the generation and analysis of the bulk and transcriptome and single-cell transcriptome data. X.W., V.C.W.N., and J.C.Y. assisted in mouse embryo harvesting and data collection. B.N. assisted in the bulk transcriptome data analysis. P.C. assisted in the single-cell transcriptome analysis. K.S.E.C., S.S., S.M., M.K., and H.H.N.

participated in the experimental design, data interpretation, and revision of the manuscript.

ACKNOWLEDGMENTS

This work was supported by grants from the Hong Kong Research Grants Council (HKU761113M, C7030-18GF, and T12-708/12-N), the Ministry of Science and Technology of the People's Republic of China: National Strategic Basic Research Program ("973") 2014CB942901, German Research Foundation FOR2722, and the S.Y. and H.Y. Cheng Professor endowment in Stem Cell Biology and Regenerative Medicine.

Received: February 26, 2018
Revised: August 14, 2019
Accepted: August 15, 2019
Published: September 12, 2019

REFERENCES

Barker, N., van Es, J.H., Kuipers, J., Kujala, P., van den Born, M., Cozijnsen, M., Haegebarth, A., Korving, J., Begthel, H., Peters, P.J., et al. (2007). Identification of stem cells in small intestine and colon by marker gene Lgr5. Nature *449*, 1003–1007.

Craig, F.M., Bentley, G., and Archer, C.W. (1987). The spatial and temporal pattern of collagens I and II and keratan sulphate in the developing chick metatarsophalangeal joint. Development *99*, 383–391.

de Lau, W., Barker, N., Low, T.Y., Koo, B.K., Li, V.S.W., Teunissen, H., Kujala, P., Haegebarth, A., Peters, P.J., van de Wetering, M., et al. (2011). Lgr5 homologues associate with Wnt receptors and mediate R-spondin signalling. Nature *476*, 293–U257.

Decker, R.S., Koyama, E., and Pacifici, M. (2014). Genesis and morphogenesis of limb synovial joints and articular cartilage. Matrix Biol. *39*, 5–10.

Decker, R.S., Um, H.B., Dyment, N.A., Cottingham, N., Usami, Y., Enomoto-Iwamoto, M., Kronenberg, M.S., Maye, P., Rowe, D.W., Koyama, E., et al. (2017). Cell origin, volume and arrangement are drivers of articular cartilage formation, morphogenesis and response to injury in mouse limbs. Dev. Biol. *426*, 56–68.

Diez-Roux, G., Banfi, S., Sultan, M., Geffers, L., Anand, S., Rozado, D., Magen, A., Canidio, E., Pagani, M., Peluso, I., et al. (2011). A high-resolution anatomical atlas of the transcriptome in the mouse embryo. PLoS Biol. *9*, e1000582.

Dowthwaite, G.P., Bishop, J.C., Redman, S.N., Khan, I.M., Rooney, P., Evans, D.J., Haughton, L., Bayram, Z., Boyer, S., Thomson, B., et al. (2004). The surface of articular cartilage contains a progenitor cell population. J. Cell Sci. *117*, 889–897.

Dy, P., Smits, P., Silvester, A., Penzo-Mendez, A., Dumitriu, B., Han, Y., de la Motte, C.A., Kingsley, D.M., and Lefebvre, V. (2010). Synovial joint morphogenesis requires the chondrogenic action of Sox5 and Sox6 in growth plate and articular cartilage. Dev. Biol. *341*, 346–359.

Gao, B., Hu, J., Stricker, S., Cheung, M., Ma, G., Law, K.F., Witte, F., Briscoe, J., Mundlos, S., He, L., et al. (2009). A mutation in Ihh that causes digit abnormalities alters its signalling capacity and range. Nature *458*, 1196–1200.

Gunnell, L.M., Jonason, J.H., Loiselle, A.E., Kohn, A., Schwarz, E.M., Hilton, M.J., and O'Keefe, R.J. (2010). TAK1 regulates cartilage and joint development via the MAPK and BMP signaling pathways. J. Bone Miner. Res. *25*, 1784–1797.

Guo, X.Z., Day, T.F., Jiang, X.Y., Garrett-Beal, L., Topol, L., and Yang, Y.Z. (2004). Wnt/beta-catenin signaling is sufficient and necessary for synovial joint formation. Genes Dev. *18*, 2404–2417.

Hartmann, C., and Tabin, C.J. (2001). Wnt-14 plays a pivotal role in inducing synovial joint formation in the developing appendicular skeleton. Cell *104*, 341–351.

Huang da, W., Sherman, B.T., and Lempicki, R.A. (2009). Systematic and integrative analysis of large gene lists using DAVID bioinformatics resources. Nat. Protoc. *4*, 44–57.

Hunter, W. (1995). Of the structure and disease of articulating cartilages. 1743. Clin. Orthop. Relat. Res. *317*, 3–6.

Kharchenko, P.V., Silberstein, L., and Scadden, D.T. (2014). Bayesian approach to single-cell differential expression analysis. Nat. Methods *11*, 740–742.

Kim, D., Langmead, B., and Salzberg, S.L. (2015). HISAT: a fast spliced aligner with low memory requirements. Nat. Methods *12*, 357–360.

Koch, M., Schulze, J., Hansen, U., Ashwodt, T., Keene, D.R., Brunken, W.J., Burgeson, R.E., Bruckner, P., and Bruckner-Tuderman, L. (2004). A novel marker of tissue junctions, collagen XXII. J. Biol. Chem. *279*, 22514–22521.

Koyama, E., Shibukawa, Y., Nagayama, M., Sugito, H., Young, B., Yuasa, T., Okabe, T., Ochiai, T., Kamiya, N., Rountree, R.B., et al. (2008). A distinct cohort of progenitor cells participates in synovial joint and articular cartilage formation during mouse limb skeletogenesis. Dev. Biol. *316*, 62–73.

Kozhemyakina, E., Zhang, M., Ionescu, A., Ayturk, U.M., Ono, N., Kobayashi, A., Kronenberg, H., Warman, M.L., and Lassar, A.B. (2015). Identification of a Prg4-expressing articular cartilage progenitor cell population in mice. Arthritis Rheumatol. *67*, 1261–1273.

Leung, C., Tan, S.H., and Barker, N. (2018). Recent advances in Lgr5(+) stem cell research. Trends Cell Biol. *28*, 380–391.

Li, L., Newton, P.T., Bouderlique, T., Sejnohova, M., Zikmund, T., Kozhemyakina, E., Xie, M., Krivanek, J., Kaiser, J., Qian, H., et al. (2017). Superficial cells are self-renewing chondrocyte progenitors, which form the articular cartilage in juvenile mice. FASEB J. *31*, 1067–1084.

Makarenkova, H.P., and Meech, R. (2012). Barx homeobox family in muscle development and regeneration. Int. Rev. Cell Mol. Biol. *297*, 117–173.

Merino, R., Macias, D., Ganan, Y., Economides, A.N., Wang, X., Wu, Q., Stahl, N., Sampath, K.T., Varona, P., and Hurle, J.M. (1999). Expression and function of Gdf-5 during digit skeletogenesis in the embryonic chick leg bud. Dev. Biol. *206*, 33–45.

Mitrovic, D.R. (1977). Development of the metatarsophalangeal joint of the chick embryo: morphological, ultrastructural and histochemical studies. Am. J. Anat. *150*, 333–347.

Nakamura, Y., Yamamoto, K., He, X., Otsuki, B., Kim, Y., Murao, H., Soeda, T., Tsumaki, N., Deng, J.M., Zhang, Z., et al. (2011). Wwp2 is

essential for palatogenesis mediated by the interaction between Sox9 and mediator subunit 25. Nat. Commun. *2*, 251.

Pryce, B.A., Brent, A.E., Murchison, N.D., Tabin, C.J., and Schweitzer, R. (2007). Generation of transgenic tendon reporters, ScxGFP and ScxAP, using regulatory elements of the scleraxis gene. Dev. Dyn. *236*, 1677–1682.

Rountree, R.B., Schoor, M., Chen, H., Marks, M.E., Harley, V., Mishina, Y., and Kingsley, D.M. (2004). BMP receptor signaling is required for postnatal maintenance of articular cartilage. PLoS Biol. *2*, 1815–1827.

Ruffner, H., Sprunger, J., Charlat, O., Leighton-Davies, J., Grosshans, B., Salathe, A., Zietzling, S., Beck, V., Therier, M., Isken, A., et al. (2012). R-Spondin potentiates Wnt/beta-catenin signaling through orphan receptors LGR4 and LGR5. PLoS One *7*, e40976.

Shwartz, Y., and Zelzer, E. (2014). Nonradioactive in situ hybridization on skeletal tissue sections. Methods Mol. Biol. *1130*, 203–215.

Shwartz, Y., Viukov, S., Krief, S., and Zelzer, E. (2016). Joint development involves a continuous influx of Gdf5-positive cells. Cell Rep. *15*, 2577–2587.

Singh, P.N.P., Yadav, U.S., Azad, K., Goswami, P., Kinare, V., and Bandyopadhyay, A. (2018). NFIA and GATA3 are crucial regulators of embryonic articular cartilage differentiation. Development *145*. https://doi.org/10.1242/dev.156554.

Soriano, P. (1999). Generalized lacZ expression with the ROSA26 Cre reporter strain. Nat. Genet. *21*, 70–71.

Storm, E.E., and Kingsley, D.M. (1999). GDF5 coordinates bone and joint formation during digit development. Dev. Biol. *209*, 11–27.

Sugimoto, Y., Takimoto, A., Akiyama, H., Kist, R., Scherer, G., Nakamura, T., Hiraki, Y., and Shukunami, C. (2013). Scx^+/$Sox9^+$ progenitors contribute to the establishment of the junction between cartilage and tendon/ligament. Development *140*, 2280–2288.

Zulch, A., Becker, M.B., and Gruss, P. (2001). Expression pattern of Irx1 and Irx2 during mouse digit development. Mech. Dev. *106*, 159–162.

Zwolanek, D., Veit, G., Eble, J.A., Gullberg, D., Ruggiero, F., Heino, J., Meier, M., Stetefeld, J., and Koch, M. (2014). Collagen XXII binds to collagen binding integrins via the novel motifs GLQGER and GFKGER. Biochem. J. *459*, 217–227.

Stem Cell Reports, Volume *13*

Supplemental Information

Lgr5 and *Col22a1* Mark Progenitor Cells in the Lineage toward Juvenile Articular Chondrocytes

Chen Feng, Wilson Cheuk Wing Chan, Yan Lam, Xue Wang, Peikai Chen, Ben Niu, Vivian Chor Wing Ng, Jia Chi Yeo, Sigmar Stricker, Kathryn Song Eng Cheah, Manuel Koch, Stefan Mundlos, Huck Hui Ng, and Danny Chan

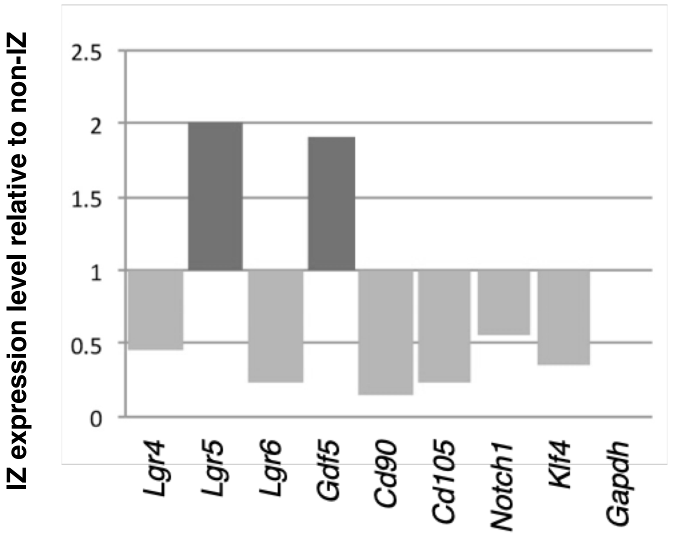

Figure S1. Quantitative PCR showed expression of Gdf5 and Lgr5 in digit interzone mRNA was extracted from the digit interzone (IZ) and surrounding non-interzone regions of a E14.5 embryo to generate cDNA for QPCR. Expression of *Gapdh* was normalized to 1.0, values higher than 1.0 indicates these genes are upregulated, vice versa. Among all the selected stem cell/progenitor markers, known marker of joint interzone *Gdf5* and novel marker identified in this paper *Lgr5* were upregulated in the interzone region.

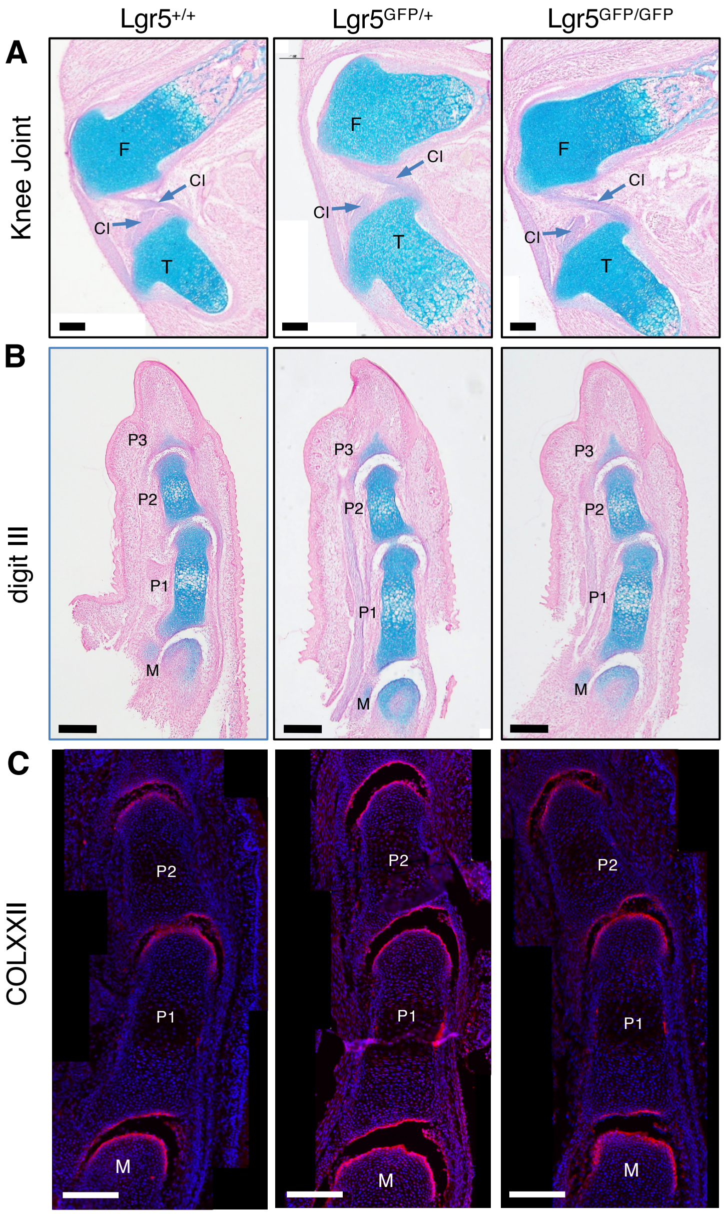

Figure S2. *Lgr5-GFP* mutant developed normal knee and digit joints
Representative histology sections stained with Alcian blue and nuclear fast red, showing no observable difference between the developing knee **(A)** and digit joints **(B)** of Lgr5$^{+/+}$, Lgr5$^{GFP/+}$ and Lgr5$^{GFP/GFP}$ E18.5 embryos (n=3 per genotype). **(C)** The articular cartilages appeared to have formed normally with the expected expression and localization of COLXXII in the superficial layer. F=femur, T=tibia, Cl=cruciate ligament, M=metacarpal, P1= proximal phalange, P2=middle phalange, P3=distal phalange. Scale bar, 200μm

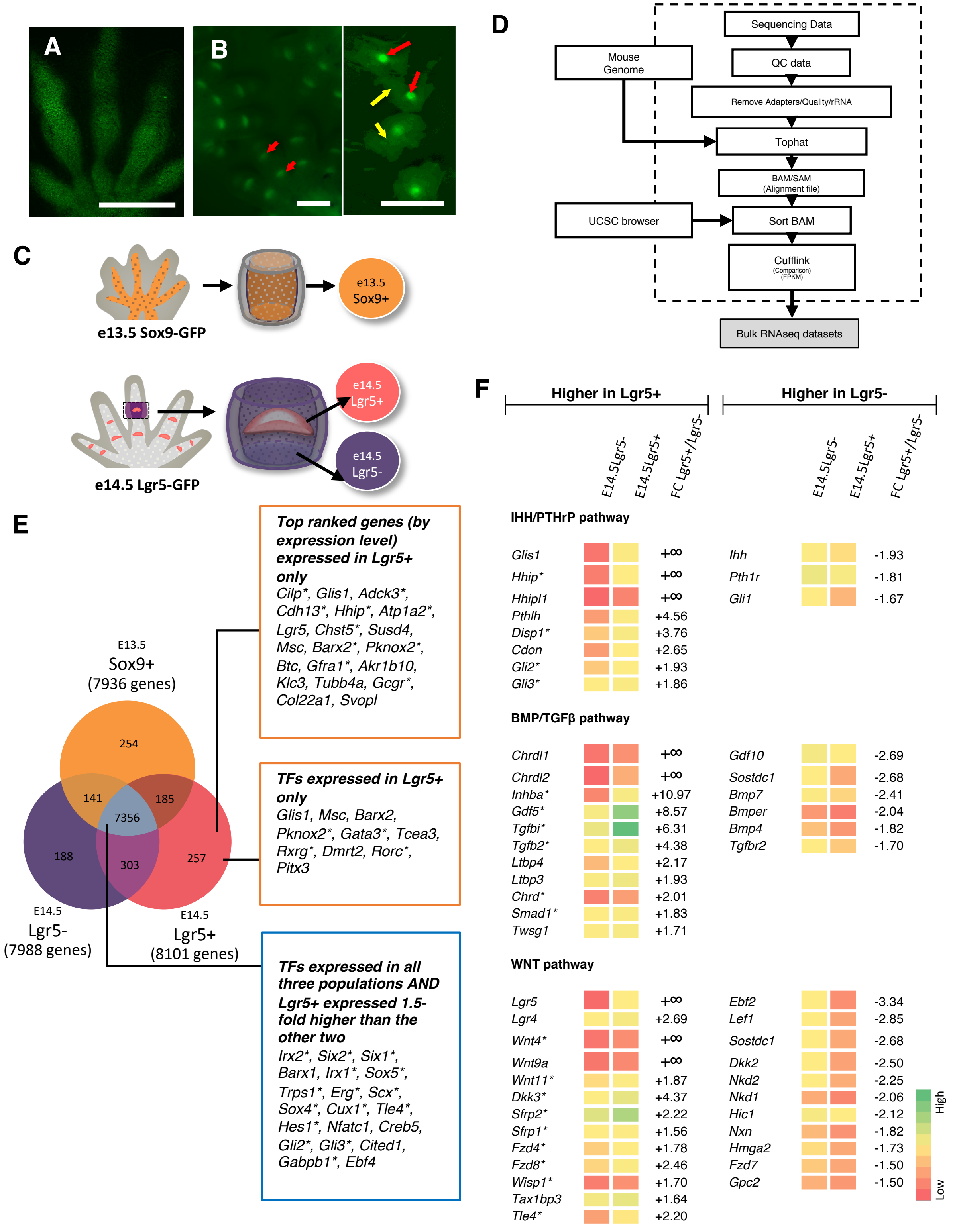

Figure S3. Bulk transcriptomic analysis of cell populations in joint development.
(A) Three population of cells isolated. One from Sox9 expressing cells in E13.5 digit of Sox9-eGFP mice, and two populations from the E14.5 interzone region of the developing digits that contains the Lgr5+ interzone (red arrows) and surrounding non-GFP region (yellow arrows) from Lgr5-GFP mice **(B)**. Scalebars, 1mm. **(C)** Diagrammatic illustration of the protocol with all cell isolated were sorted for the appropriate population of cells for subsequent for RNA-seq for the generating the three datasets. **(D)** Workflow for bulk RNAseq data processing. **(E)** Venn diagram showing the number of genes identified, and overlaps between datasets. Genes with higher expression in Lgr5+ population are shown on the right. **(F)** Heat maps depicting comparative gene expression levels of the top genes involved in specified molecular pathways. + Fold change (FC) indicates an increase and -FC for decrease in Lgr5+ cells. "+∞ " are given to indicate genes that are expressed only in Lgr5+ cells. Genes with expression data available in the Eurexpress database are marked with an asterisk.
Related to figure 3.

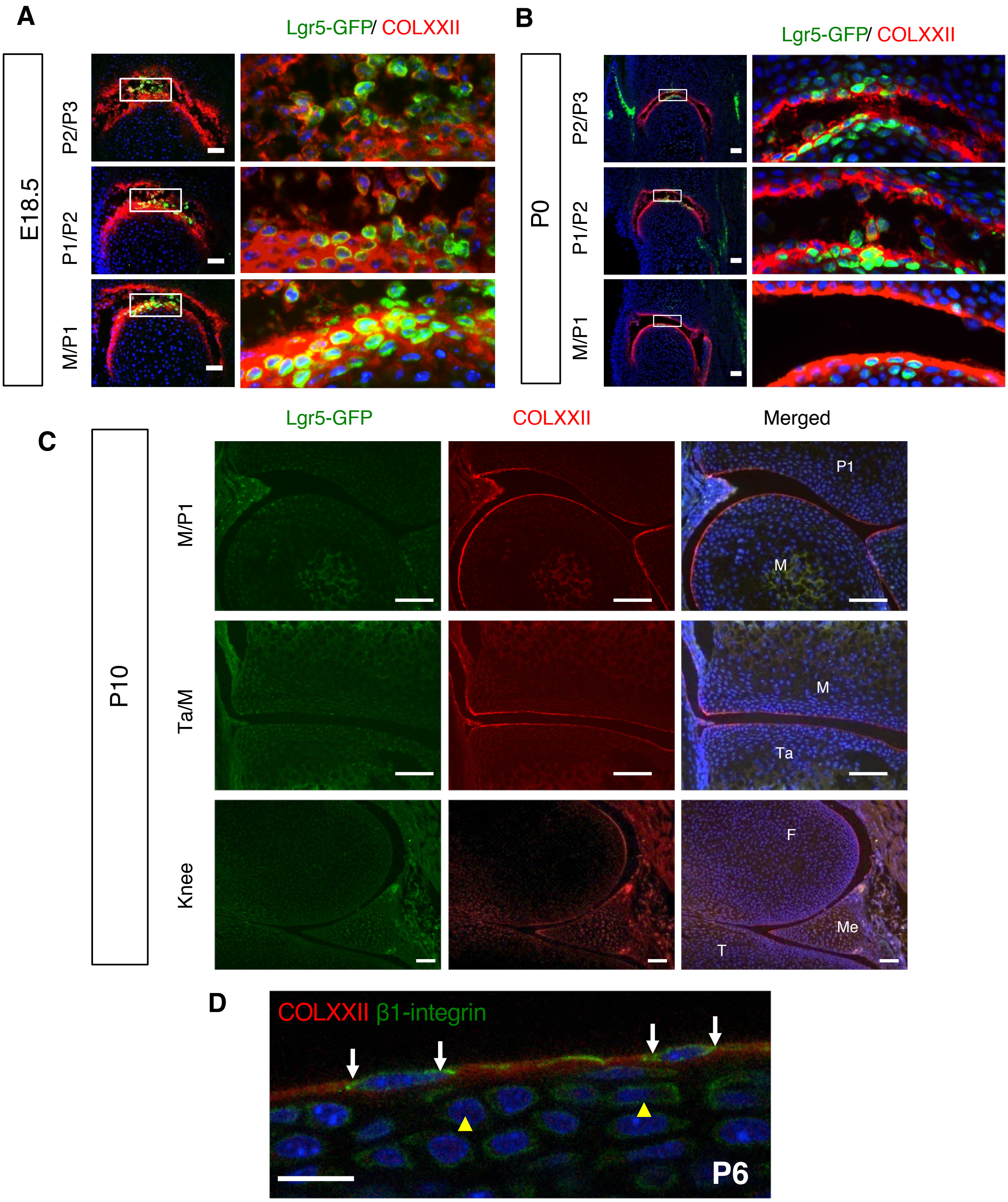

Figure S4. Expression of Lgr5 and COLXXII in phalangeal and knee joints.

Co-localization of Lgr5 (Green) and COLXXII (Red) in E18.5 **(A)** and newborn (P0) **(B)** digit joints. **(C)** Expression in M/P1, Ta/M and Knee joints at P10. M, metatarsal. P1, proximal phalange. P2, middle phalange. P3, distal phalange. Ta/M, tarsometatarsal joint. **(D)** Cells embedded in the COLXXII layer are flat compare to the round chondrocytes in the deeper zones (yellow arrow) of P6 mouse. Focal adhesions (β1-integrin) are found at both sides of the cells where they are in direct contact with the COLXXII matrix (white arrows). Scale bars in (A-C), 100μm and in (D), 10μm. **Related to figure 3.**

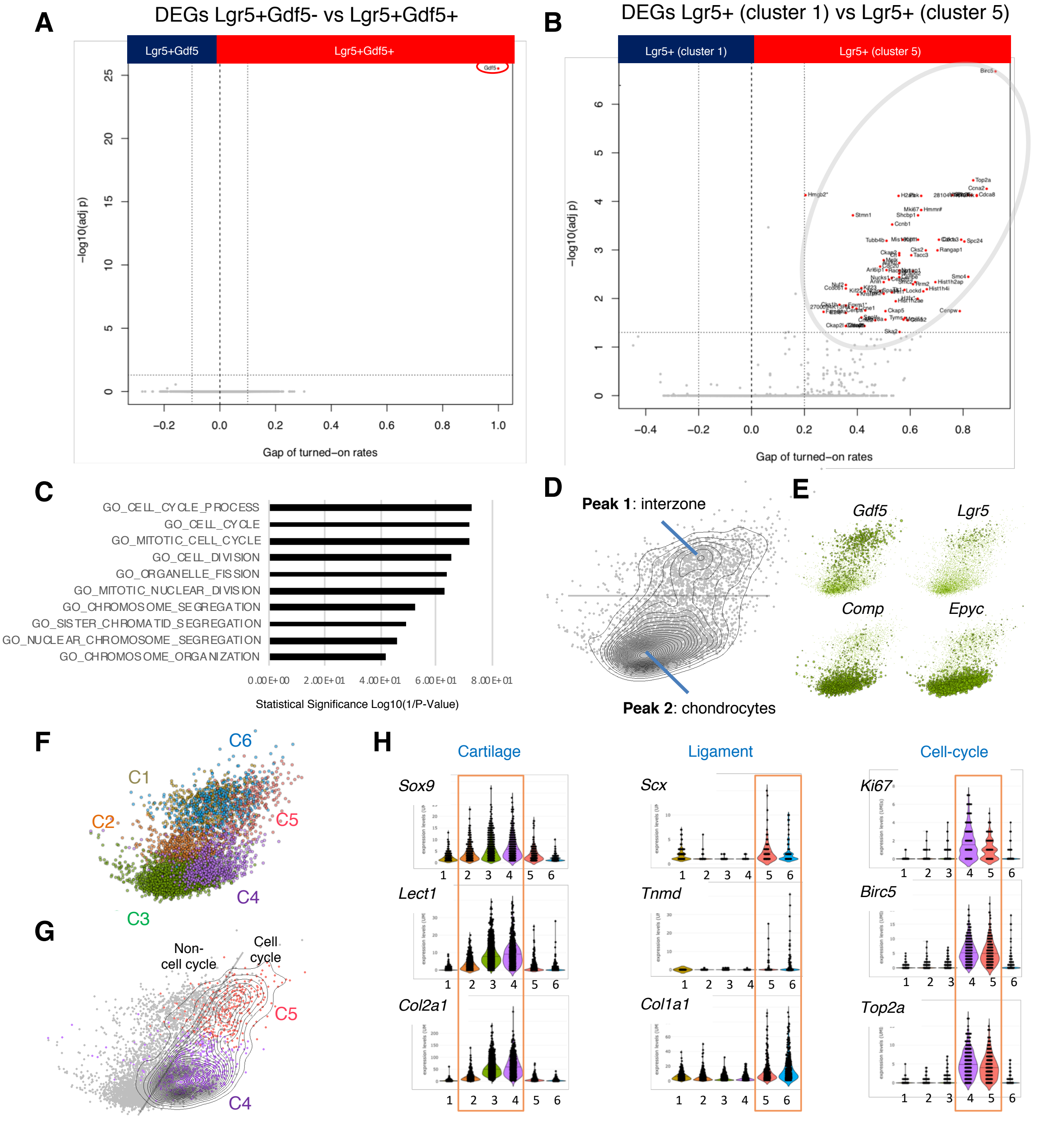

Figure S5 Additional information for single cell analysis

(A) Volcano plot showing *Gdf5* (red circle) is the only differential expressing gene (DEG) between *Lgr5+/Gdf5-* and *Lgr5+/Gdf5+* subpopulations, and these cells are essential identical. **(B)** Volcano plot shows DEGs between Lgr5+ cells from cluster 1 and cluster 5, also show they are the same cells, differ only as proliferating cells in cluster 5. **(C)** GO term analysis of the DEGs from panel (B) (circled). (d-g) Principle component analysis (PCA) for the 5,460 E14.5 knee joint cells. **(D)** A contour plot showing the density of cells, two major peaks are found. **(E)** Peak 1 corresponds to interzone cells expressing *Gdf5* and *Lgr5*, and peak 2 as non-interzone expressing mature chondrocyte markers, *Comp* and *Epyc*. **(F)** t-SNE clusters 1, 2, 5, and 6 are located in peak 1, whereas clusters 3 and 4 are in peak 2, consistent with the 2D t-SNE finding. **(G)** Contour plot of the cell cycle genes showing the existence of proliferating subpopulation of cell in the interzone (cluster 5) and non-interzone (cluster 4) clusters. **(H)** Violin plots showing the expression profiles of representative cartilage, ligament and cell cycle genes in each cluster supporting their identities/signatures. **Related to figure 4.**

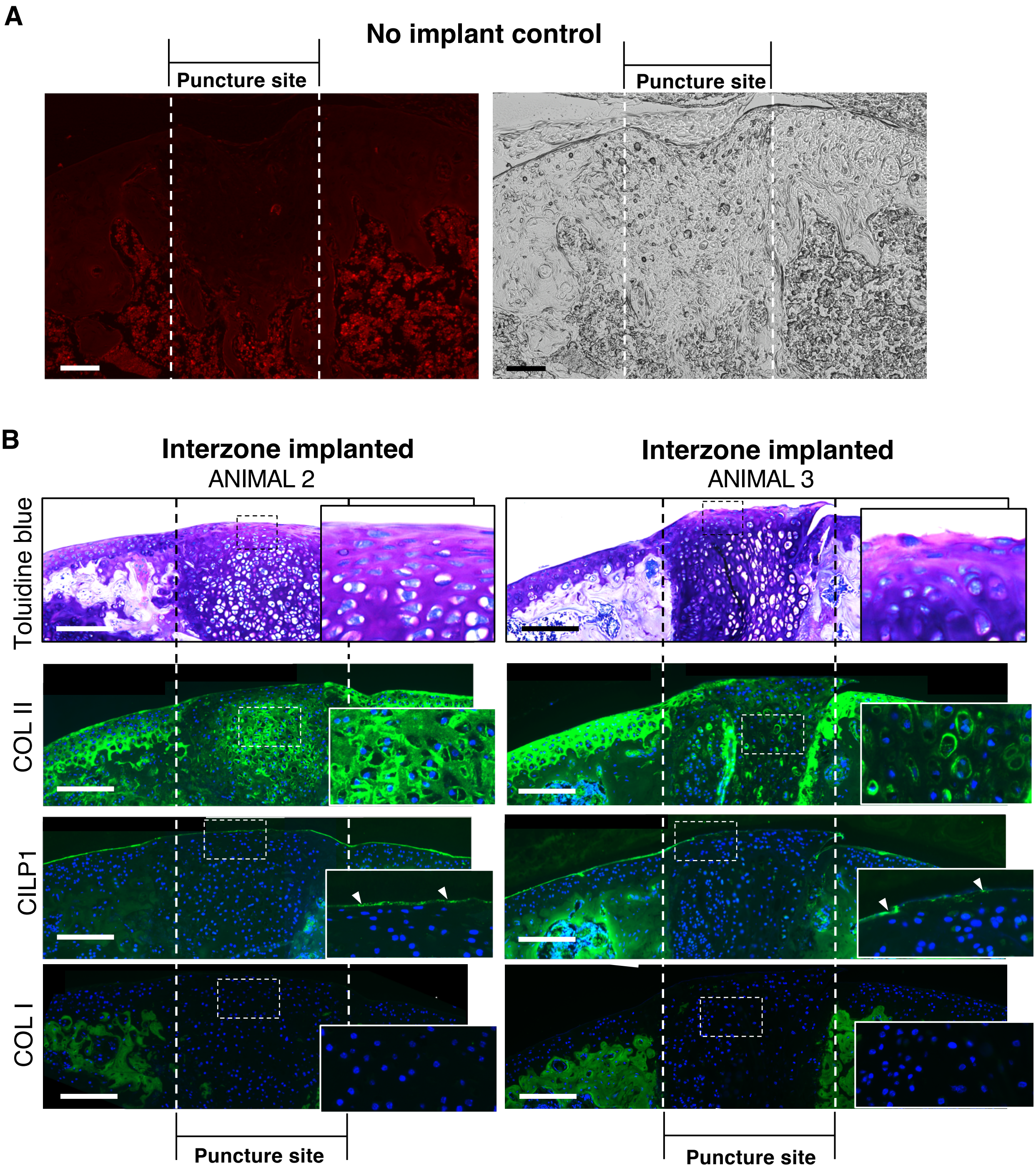

Figure S6. ***Lgr5*+ interzone tissue transplantation for repairing of a cartilage lesion**

(A) No tdTomato+ cells was detected in the puncture site without interzone tissue insert. Bright-field image of the same region was shown on the right. **(B)** Analysis of two additional animals receiving interzone tissue transplantation. Similar positive outcomes among all three operated animals, with differentiation of the interzones to chondrocytes expressing COL II, reestablishment of the superficial layer (CILP1 expressing), and suppressing COL I expression and fibrotic tissue. Scalebars, 50μm. **Related to figure 6.**

Table S1. Genes expressed by only one population (top 100 ranked by level) (Digit bulk)

	Expressed in Sox9+ only (FPKM>5)				Expressed in Lgr5- only (FPKM>5)				Expressed in Lgr5+ only (FPKM>5)			
	**Gene**	**e13.5 Sox9+**	**e14.5 Lgr5-**	**e14.5 Lgr5+**	**Gene**	**e13.5 Sox9+**	**e14.5 Lgr5-**	**e14.5 Lgr5+**	**Gene**	**e13.5 Sox9+**	**e14.5 Lgr5-**	**e14.5 Lgr5+**
**1**	*Epyc*	**48.82**	0.63	1.2	*Alas2*	0.86	**25.41**	1.16	*Cilp*	0.2	0.75	**58.03**
**2**	*Smpd3*	**30.66**	0.29	0.19	*Foxf2*	2.52	**20.2**	2.85	*Glis1*	4.33	3.54	**52.57**
**3**	*Panx3*	**27.67**	1.16	1.14	*Stra6*	4.58	**18.26**	4.13	*Adck3*	0.99	2.62	**35.46**
**4**	*Epha2*	**20.86**	4.24	4.68	*Cpa3*	0.81	**15.64**	2.79	*Cdh13*	2.91	2.97	**35.39**
**5**	*Frzb*	**20.82**	1.77	2.12	*Sst*	0.23	**15.56**	4.07	*Hhip*	2.97	4.94	**30.22**
**6**	*Foxd1*	**19.47**	3.43	0.71	*Cd74*	1.82	**12.69**	4.52	*Atp1a2*	3.4	1.89	**27.54**
**7**	*Cxcl10*	**18.67**	1.73	3.35	*Zfhx4*	4.26	**12.46**	4.66	*Lgr5*	3.48	1.36	**26.75**
**8**	*Pltp*	**17.52**	3.89	2.93	*Cma1*	0.39	**12.41**	2.26	*Chst5*	4.06	3.01	**26.54**
**9**	*Hoxc10*	**17.02**	0.21	0.37	*Ablim1*	3.74	**12.18**	4.9	*Susd4*	3.6	4.89	**22.94**
**10**	*Tnfaip3*	**16.98**	4.13	4.71	*S100a8*	0	**11.5**	0.86	*Msc*	2.18	1.28	**22.79**
**11**	*Peg10*	**14.87**	3.71	2.4	*Grp*	1.24	**11.19**	4.11	*Barx2*	1.27	0.25	**20.41**
**12**	*Spp1*	**14.72**	2.5	2.13	*Snca*	0.69	**9.95**	1.52	*Pknox2*	3.74	1.42	**19.37**
**13**	*Sgms2*	**14.54**	3.67	3.36	*Fgf2*	4.52	**9.61**	4.19	*Btc*	0.46	0.56	**19.22**
**14**	*Chac1*	**14.27**	0.64	1.38	*Angpt2*	0.78	**9.45**	1.97	*Gfra1*	2.9	3.67	**18.25**
**15**	*Ndufa4l2*	**14.12**	2.13	3.35	*Kcp*	4.12	**9.03**	4.84	*Akr1b10*	3.87	1.78	**18.06**
**16**	*Chst11*	**14.06**	4.4	4.75	*Arhgdig*	4.34	**9.01**	4.21	*Klc3*	3.59	4.05	**17.07**
**17**	*Ptgs2*	**13.6**	1.04	1.41	*Axin2*	2.12	**8.94**	3.13	*Tubb4a*	4.29	4.63	**16.12**
**18**	*Mpzl2*	**13.06**	3.79	4.67	*Cd52*	0	**8.86**	2.87	*Gcgr*	3.2	1.77	**15.97**
**19**	*Edil3*	**12.86**	3.73	4.66	*Kcnk2*	3.13	**8.79**	4.11	*Col22a1*	1.5	1.93	**15.82**
**20**	*S100b*	**12.1**	2.56	2.62	*Stmn2*	2.71	**8.7**	1.51	*Svopl*	1.11	1	**15.72**
**21**	*Foxo6*	**12.07**	4.31	3.36	*Dbx2*	1.4	**8.68**	0.74	*Vaultrc5*	4.63	2.57	**15.48**
**22**	*Sytl2*	**12.03**	2.32	4.18	*Alox5ap*	1.35	**8.64**	2.61	*Pde2a*	2.11	2.81	**15.23**
**23**	*C1galt1*	**11.68**	3.47	4.69	*Nxnl2*	2.17	**8.62**	3.73	*Ppp1r1b*	2	2.62	**14.44**
**24**	*Cgref1*	**11.62**	4.64	4.25	*Srgn*	0.45	**8.58**	2.88	*Sntb1*	1.77	2.9	**13.36**
**25**	*Pmaip1*	**11.61**	2.98	2.64	*Cmpk2*	3.86	**8.56**	4.93	*Thbs4*	2.09	2.94	**12.9**
**26**	*Tcf15*	**11.41**	4.13	3.25	*F11r*	3.94	**8.27**	3.13	*Krt5*	4.61	1.24	**12.62**
**27**	*Saa1*	**11.33**	2.08	4.06	*Kif26a*	3.06	**8.07**	1.13	*Lrrn3*	2.5	1.52	**12.38**
**28**	*Hck*	**10.72**	4.42	1.37	*Gstm6*	2.7	**8.06**	3.74	*Tnnc1*	1.83	1.07	**12.27**
**29**	*Xylt1*	**10.7**	4.44	3.26	*Heph*	2.99	**7.92**	3.35	*Ephx1*	1.74	2.72	**12.15**
**30**	*Entpd6*	**10.53**	4.55	4.95	*H2-T22*	2.59	**7.91**	4.31	*Stc1*	3.61	4.5	**11.55**
**31**	*Col15a1*	**10.4**	3.93	2.15	*Il17rd*	2.63	**7.89**	2.76	*Cer1*	2.53	0.03	**11.04**
**32**	*Magel2*	**10.39**	4.97	3.5	*Gpr124*	4.32	**7.8**	2.74	*Ptpro*	0.68	0.76	**10.97**
**33**	*Ifitm5*	**10.38**	2.22	1.74	*Fbxl19*	4.86	**7.78**	4.63	*Matk*	2.46	2.32	**10.56**
**34**	*Bmp5*	**10.25**	4.5	2.69	*Tmtc2*	2.81	**7.77**	2.37	*Acta2*	2.64	3.18	**10.21**
**35**	*E2f7*	**9.81**	4.32	3.38	*Lgals7*	2.52	**7.75**	3.95	*Ppp1r1a*	3.75	2.33	**9.98**
**36**	*Foxa3*	**9.6**	2.47	3.36	*Raver1-fdx1l*	3.61	**7.36**	3.59	*Kcnma1*	4.52	2.85	**9.71**
**37**	*Ssfa2*	**9.58**	4.32	4.96	*Adamts9*	3.79	**7.33**	3.19	*Sh2b2*	3.44	4.96	**9.6**
**38**	*Clec11a*	**9.43**	2.23	1.37	*Nek8*	4.51	**7.31**	4.84	*Glrb*	2.27	4.98	**9.5**
**39**	*Eif3j*	**9.34**	0.33	0.66	*Hdc*	0.85	**7.31**	0.71	*Des*	1.17	1.61	**9.33**
**40**	*Serinc5*	**9.13**	2.57	2.94	*Ngfr*	0.67	**7.28**	3.72	*Camk1g*	3.22	4.51	**9.32**
**41**	*Ncoa7*	**9.03**	2.27	2.52	*Scara5*	1.44	**7.15**	3.18	*Pitpnm1*	4.26	4.74	**9.26**
**42**	*Hoxa5*	**8.96**	2.36	1.97	*Pdxk*	4.82	**7.12**	4.5	*Gdf1*	3.37	3.67	**9.1**
**43**	*Dlc1*	**8.89**	3.62	3.41	*Kctd1*	4.39	**7.12**	3.9	*Krtdap*	0.62	0.19	**9.02**
**44**	*E2f8*	**8.67**	2.56	3.61	*Cav1*	2.19	**6.92**	3.6	*Kcnj15*	0.4	0.47	**9.02**
**45**	*Otor*	**8.65**	3.95	2.44	*Tubb3*	2.06	**6.9**	1.24	*Ccl4*	1.41	4.75	**8.88**
**46**	*Stil*	**8.53**	3.47	3.27	*Arhgap39*	4.5	**6.89**	3.82	*Sfn*	3.54	1.38	**8.86**
**47**	*Avpr1a*	**8.45**	1.25	0.8	*Lypd6*	2.57	**6.71**	3.31	*Sema3e*	3.21	3.2	**8.8**
**48**	*Fndc3c1*	**8.39**	2.26	2.93	*Lbh*	4.96	**6.7**	2.52	*Myod1*	0.46	1.69	**8.77**

**49**	*Frat1*	**8.29**	2.48	4.36	*Slc25a14*	3.08	**6.7**	4.78	*Smtnl2*	3.1	4.07	**8.64**
**50**	*Armcx5*	**8.25**	3.75	4.53	*Ophn1*	4.37	**6.68**	4.33	*Pf4*	1.69	3.34	**8.57**
**51**	*Nol12*	**8.23**	4.59	4.21	*Adamts17*	3.42	**6.67**	3.66	*Snap91*	2.29	1.93	**8.43**
**52**	*Ptger4*	**8.16**	2.88	2.04	*Mterfd3*	3.9	**6.62**	4.78	*Dcx*	2.93	2.18	**8.41**
**53**	*Ttll3*	**8.13**	4.77	4.21	*Rev3l*	4.38	**6.58**	4.67	*Fam159a*	3.68	3.42	**8.39**
**54**	*Dclre1c*	**8.02**	2.77	4.02	*Acsf3*	3.84	**6.54**	3.97	*Accs*	2.66	3.37	**8.32**
**55**	*Stk17b*	**8**	2.3	1.7	*Stom*	2.8	**6.54**	4.95	*Gata3*	3.49	0.84	**8.3**
**56**	*Hist1h2bn*	**8**	3.32	3.49	*Ank2*	3.39	**6.48**	4.86	*Myl1*	1.83	2.24	**8.28**
**57**	*Omd*	**7.96**	3.38	3.72	*Bcl2l11*	3.94	**6.42**	4.94	*Slc25a23*	2.77	4.17	**8.13**
**58**	*Pcdh1*	**7.9**	0.67	2.57	*Ankrd44*	2.78	**6.42**	3.49	*Tcea3*	1.68	4.53	**8.08**
**59**	*Npat*	**7.88**	4.84	4.74	*Nkain1*	3.17	**6.4**	3.93	*Doc2g*	4.83	4.62	**7.9**
**60**	*Cbr2*	**7.86**	4.12	3.25	*Ptges*	3.93	**6.39**	3.44	*Spon2*	0.21	2.12	**7.89**
**61**	*Hspg2*	**7.84**	3.74	2.28	*Kit*	2.35	**6.38**	2.5	*Abcg4*	3.87	4.93	**7.88**
**62**	*Pygl*	**7.81**	3.93	3.2	*Mgat3*	3.77	**6.35**	3.81	*Adamts14*	3.42	2.32	**7.81**
**63**	*Krtcap3*	**7.75**	2.34	4.88	*Fzd10*	1.6	**6.33**	1.17	*Krt15*	2.78	1.78	**7.81**
**64**	*Shroom3*	**7.72**	4.68	4.46	*Slitrk5*	3.45	**6.31**	3.4	*Dleu2*	1.11	3.16	**7.77**
**65**	*Hist1h3g*	**7.67**	4.3	2.68	*Ddx23*	4.78	**6.29**	4.63	*Cntfr*	2.19	1.1	**7.71**
**66**	*Scd1*	**7.62**	3.42	1.66	*Camk2g*	4.35	**6.26**	4.27	*Phactr1*	1.35	1.55	**7.69**
**67**	*Ung*	**7.59**	3.32	2.78	*Fap*	1.5	**6.26**	3.85	*Fbp2*	4.11	1.92	**7.65**
**68**	*Nppc*	**7.59**	3.69	3.88	*Rhobtb2*	4.82	**6.25**	4.68	*Ano1*	2.91	2.32	**7.62**
**69**	*Baiap2l1*	**7.58**	2.65	2.78	*Tgfbr3*	4.87	**6.22**	3.44	*Ehbp1l1*	3.16	3.34	**7.59**
**70**	*Tgfa*	**7.57**	2.66	1.99	*Atxn7l1*	4.59	**6.19**	3.89	*Uap1l1*	3.42	4.25	**7.53**
**71**	*Adamts3*	**7.53**	4.01	2.9	*Wbp7*	4.39	**6.12**	4.97	*Wdr67*	4.62	4.69	**7.41**
**72**	*Prkg2*	**7.48**	1.73	1.22	*Iqgap2*	4.99	**6.1**	4.34	*Tmem117*	1.95	1.4	**7.39**
**73**	*Orc1*	**7.4**	3.33	3.57	*Sdpr*	2.35	**6.09**	3.2	*Dynlt3*	4.2	4.23	**7.38**
**74**	*Aldoc*	**7.38**	3.34	4.4	*Zfp322a*	4.84	**6.06**	4.9	*Tle6*	3.26	4.42	**7.36**
**75**	*Foxf1a*	**7.37**	0.58	1.26	*Slc2a4*	3.46	**6.06**	2.19	*Ramp1*	0.56	0.62	**7.33**
**76**	*Cenpk*	**7.36**	4.04	4.36	*Pear1*	4.79	**6.04**	3.57	*Rxrg*	4.78	2.31	**7.22**
**77**	*Fam180a*	**7.35**	1.15	2.41	*Mtss1*	3.04	**6.03**	4.92	*St7l*	4.32	4.72	**7.18**
**78**	*Tgm2*	**7.34**	3.85	3.84	*Traf5*	4.46	**6.01**	4.74	*Wnt9a*	3.13	3.77	**7.17**
**79**	*Esco2*	**7.34**	4.01	4.35	*Fgf10*	2.45	**6**	2.21	*Tesk2*	4.7	4.25	**7.16**
**80**	*Hip1r*	**7.33**	3.6	4.68	*Gdf11*	3.4	**5.98**	4.88	*Coq10a*	4.14	4.78	**7.16**
**81**	*Slc19a2*	**7.32**	4.28	4.71	*Txnrd2*	3.51	**5.95**	3.92	*Fam20c*	3.93	3.24	**7.16**
**82**	*Cenpf*	**7.32**	3.48	4.93	*Tspan11*	1.58	**5.95**	3.57	*Rarres2*	3.09	3.66	**7.16**
**83**	*Pdlim1*	**7.29**	3.41	2.27	*Kpna6*	4.89	**5.94**	4.26	*Pip4k2a*	3.77	3.38	**7.12**
**84**	*Per2*	**7.28**	2.93	4.73	*Fert2*	4.55	**5.9**	4.66	*Dmrt2*	1.31	2.73	**7.06**
**85**	*Ascc3*	**7.27**	4.03	4.96	*Zfp397*	4.26	**5.9**	4.14	*Nt5e*	2.7	1.32	**7.02**
**86**	*St6galnac2*	**7.25**	4.83	3.19	*Smcr8*	4.69	**5.89**	3.4	*Tagln*	3.33	2.95	**7.01**
**87**	*Scn1b*	**7.22**	2.19	2.08	*Gng8*	3.13	**5.88**	3.46	*Col13a1*	0.75	4.35	**6.98**
**88**	*Grem1*	**7.16**	1.11	2.71	*Sall1*	2.8	**5.88**	2.77	*Aard*	3.16	3.38	**6.97**
**89**	*Nptx2*	**7.12**	1.28	0.73	*Nlrx1*	2.62	**5.87**	4.8	*Tesc*	1.57	0.78	**6.95**
**90**	*Usp2*	**6.93**	4.18	3.64	*Prmt10*	4.21	**5.86**	4.73	*Shq1*	4.9	4.87	**6.91**
**91**	*Gpd2*	**6.93**	4.65	4.22	*Tcf7*	2.91	**5.86**	3.27	*Ell3*	3.03	2.34	**6.91**
**92**	*Snhg4*	**6.91**	3.3	2.85	*Slc22a5*	3.73	**5.84**	4.4	*C1qa*	0.31	0.86	**6.91**
**93**	*Isl1*	**6.86**	0.14	0.02	*Ptch2*	2.46	**5.83**	1.86	*Tmem175*	4.76	4.96	**6.87**
**94**	*Lingo1*	**6.78**	3.68	2.82	*Prr5l*	1.38	**5.83**	1.75	*Dner*	0.57	1.67	**6.83**
**95**	*Pcp4*	**6.75**	2.28	1.82	*Tyk2*	4.38	**5.79**	4.23	*Apba2*	3.22	3.57	**6.81**
**96**	*Fam84b*	**6.75**	2.9	3.26	*Cdc42bpa*	4.25	**5.75**	4.38	*Ppp1r3g*	3.54	1.43	**6.79**
**97**	*Dlx3*	**6.69**	3.11	1.26	*Srd5a1*	4.44	**5.73**	4.28	*Dcaf6*	3.38	3.82	**6.78**
**98**	*Rfx1*	**6.68**	4.95	3.69	*Ccdc28a*	3.3	**5.73**	3.59	*Igsf11*	2.7	1.46	**6.77**
**99**	*Arl4c*	**6.67**	2.97	3.03	*Pcnx*	4.42	**5.72**	4.81	*Krt14*	4.43	0.54	**6.76**
**100**	*Foxn2*	**6.64**	3.72	4.97	*Hnrnpul2*	4.27	**5.69**	3.96	*Fbp1*	1.5	1.46	**6.76**

Table S2. Transcription factors expressed in specific cell population (Bulk transcriptome of digit joint)

Gene	e13.5 Sox9+	e14.5 Lgr5- (FPKM)	e14.5 Lgr5+
**Transcription factors expressed in Lgr5+ only**			
Glis1	4.33	3.54	**52.57**
Msc	2.18	1.28	**22.79**
Barx2	1.27	0.25	**20.41**
Pknox2	3.74	1.42	**19.37**
Gata3	3.49	0.84	**8.3**
Tcea3	1.68	4.53	**8.08**
Rxrg	4.78	2.31	**7.22**
Dmrt2	1.31	2.73	**7.06**
Rorc	1.03	0.62	**6.02**
Pitx3	0.79	1.01	**5.71**
**Transcription factors expressed in Lgr5- only**			
Foxf2	2.52	**20.2**	2.85
Dbx2	1.4	**8.68**	0.74
Lbh	4.96	**6.7**	2.52
Ptges	3.93	**6.39**	3.44
Wbp7	4.39	**6.12**	4.97
Rfx5	4.76	**5.54**	4
Tcf20	3.21	**5.48**	3.03
**Transcription factors expressed in Sox9+ only**			
Foxd1	**19.47**	3.43	0.71
Hoxc10	**17.02**	0.21	0.37
Tnfaip3	**16.98**	4.13	4.71
Foxo6	**12.07**	4.31	3.36
Tcf15	**11.41**	4.13	3.25
Foxa3	**9.6**	2.47	3.36
Hoxa5	**8.96**	2.36	1.97
Foxf1a	**7.37**	0.58	1.26
Pdlim1	**7.29**	3.41	2.27
Per2	**7.28**	2.93	4.73
Rfx1	**6.68**	4.95	3.69
Foxn2	**6.64**	3.72	4.97
Pou3f3	**6.58**	1.83	2.45
Zim1	**6.44**	1.13	1.16
Per1	**6.41**	3.16	2.64
Hoxc9	**6.32**	0.06	0.34
Meox1	**6.03**	1.63	3.26
Nkx6-2	**5.41**	4.07	4

Table S3. Transcription factors expressed in all three populations AND Lgr5+ expressed 1.5-fold higher than the other two populations (Bulk transcriptome of digit joint)

Gene	e13.5 Sox9+	e14.5 Lgr5- (FPKM)	e14.5 Lgr5+	Fold change Lgr5+/Lgr5-	Fold change Lgr5+/Sox9+
Irx2	9.97	5.84	57.21	+9.80	+5.74
Six2	50.34	53.86	277.21	+5.15	+5.51
Six1	16.24	9.49	37.33	+3.93	+2.30
Barx1	55.41	23.81	92.04	+3.87	+1.66
Irx1	15.6	11.76	43.98	+3.74	+2.82
Sox5	21.32	10.02	35.08	+3.50	+1.65
Trps1	15.71	12.68	33.33	+2.63	+2.12
Erg	10.34	9.99	25.21	+2.52	+2.44
Scx	42.52	80.56	194.54	+2.41	+4.58
Sox4	416.76	318.43	701.26	+2.20	+1.68
Cux1	47.5	33.43	73.42	+2.20	+1.55
Tle4	14.27	11.65	25.58	+2.20	+1.79
Hes1	298.41	284.69	604.86	+2.12	+2.03
Nfatc1	52.48	39.2	80.79	+2.06	+1.54
Creb5	19.1	25.8	49.93	+1.94	+2.61
Gli2	13	19.73	38.16	+1.93	+2.94
Gli3	22.69	32.62	60.75	+1.86	+2.68
Cited1	10.79	11.28	19.14	+1.70	+1.77
Gabpb1	30.91	29.95	50.67	+1.69	+1.64
Ebf4	7.67	11.14	18.09	+1.62	+2.36

Table S4. Genes involved in molecular signaling pathways, cell-matrix contact, ECM degradation and organisation (Bulk transcriptome of E14.5 digit joint)

Gene	E14.5 Lgr5- (FPKM)	E14.5 Lgr5+ (FPKM)	Fold change relative to Lgr5-
**IHH/PTHrP pathway**			
Glis1	3.54	52.57	+∞
Hhip	4.94	30.22	+∞
Hhipl1	0.92	6.36	+∞
Pthlh	11	50.11	+4.56
Disp1	19.78	74.28	+3.76
Cdon	10.93	28.96	+2.65
Gli2	19.73	38.16	+1.93
Gli3	32.62	60.75	+1.86
Ihh	45.36	23.54	-1.93
Pth1r	131.09	72.3	-1.81
Gli1	26.77	16.03	-1.67
**BMP/TGFβ pathway**			
Chrdl1	3.52	8.64	+∞
Chrdl2	0.85	14.32	+∞
Inhba	6.76	74.18	+10.97
Gdf5	71.72	614.83	+8.57
Tgfbi	136.76	863.5	+6.31
Tgfb2	27.44	120.3	+4.38
Ltbp4	16.72	36.21	+2.17
Ltbp3	61.32	118.37	+1.93
Chrd	5.69	11.43	+2.01
Smad1	32.44	59.33	+1.83
Twsg1	43.31	73.92	+1.71
Gdf10	86.28	32.02	-2.69
Sostdc1	36.56	13.65	-2.68
Bmp7	52.52	21.81	-2.41
Bmper	10.35	5.08	-2.04
Bmp4	18.37	10.07	-1.82
Tgfbr2	33.63	19.82	-1.70
**WNT pathway**			
Lgr5	1.36	26.75	+∞
Lgr4	28.27	76.06	+2.69
Wnt4	3.52	8.09	+∞
Wnt9a	3.77	7.17	+∞
Wnt11	23.2	43.28	+1.87
Dkk3	37.72	164.84	+4.37
Sfrp2	203.06	450.71	+2.22
Sfrp1	28.5	44.32	+1.56
Fzd4	20.58	36.73	+1.78
Fzd8	21.71	53.42	+2.46
Wisp1	5.11	8.68	+1.70
Tax1bp3	92.74	151.9	+1.64
Tle4	11.65	25.58	+2.20
Ebf2	27.59	8.27	-3.34
Lef1	36.27	12.73	-2.85
Sostdc1	36.56	13.65	-2.68
Dkk2	30.03	11.99	-2.50
Nkd2	32.47	14.43	-2.25
Nkd1	13.91	6.74	-2.06
Hic1	78.08	36.77	-2.12
Nxn	16.76	9.19	-1.82
Hmga2	23.57	13.6	-1.73
Fzd7	19.6	13.08	-1.50
Gpc2	15.06	10.06	-1.50

Gene	E14.5 Lgr5- (FPKM)	E14.5 Lgr5+ (FPKM)	Fold change relative to Lgr5-
**Cell surface markers and receptors**			
Cd9	105	296.73	+2.83
Cd200	6.66	14.9	+2.24
Cd83	22.25	45.54	+2.05
Cd47	22.63	37.49	+1.66
Cd52	8.86	2.87	-∞
Cd74	12.69	4.52	-∞
Cd248	46.41	21.55	-2.15
Cd82	10.93	6.35	-1.72
Cdh13	2.97	35.39	+∞
Itga11	16.04	81.87	+5.10
Itga5	29.83	52.72	+1.77
Itgav	9.39	5.39	+1.37
Itga9	8.32	11.38	+1.05
Itgb1	92.38	97.13	+1.05
Itgb5	70.15	73.66	-2.02
Cdh3	33.83	16.78	-1.62
Cdh2	21.39	13.23	-1.76
Ddr2	15.06	8.55	-1.74
Itga6	5.78	5.44	-1.06
**Enzymes for ECM degradation**			
Adamts14	2.32	7.81	+∞
Adamtsl2	6.99	14.82	+2.12
Timp3	49.55	85.52	+1.73
Ctsf	24.52	36.84	+1.50
Chst10	10.7	6.78	-1.58
Chst15	7.46	3.17	-∞
Mmp11	124.76	77.61	-1.61
**ECM components**			
Cilp	0.75	58.03	+∞
Col22a1	1.93	15.82	+∞
Thbs4	2.94	12.9	+∞
Spon2	2.12	7.89	+∞
Tgfbi	136.76	863.5	+6.31
Fmod	24.45	134.56	+5.50
Col8a1	6.86	22.27	+3.25
Dcn	35.98	115.28	+3.20
Efemp1	12.14	34.69	+2.86
Col9a2	216.81	479.99	+2.21
Ltbp4	16.72	36.21	+2.17
Col9a3	206.32	440.71	+2.14
Col6a1	82.63	167.27	+2.02
Ltbp3	61.32	118.37	+1.93
Vcan	31.28	56.73	+1.81
Wisp1	5.11	8.68	+1.70
Col9a1	184.09	302.35	+1.64
Col11a1	104.96	169.86	+1.62
Col2a1	1400.84	2253.09	+1.61
Postn	52.87	81.99	+1.55
Emilin1	159.14	244.21	+1.53
Fbln2	159.5	243.39	+1.53
Prg4	2.36	1.14	not exp. in both
Comp	15.75	3.9	-∞
Emilin2	18.61	6.65	-2.80
Col23a1	25.86	10.96	-2.36
Matn1	93.79	41.46	-2.26
Fbn1	26.53	12.34	-2.15
Col4a1	50.96	28.98	-1.76
Fbln5	21.76	13.65	-1.59
Lox	47.87	31.36	-1.53

+∞ and -∞ indicate genes that are not expressed in Lgr5- and Lgr5+ respectively, fold change is not applicable.

Table S5. Details and parameters for single cell transcriptome sequencing and analysis. Related to Figures 4 and 5.

**Sequencing and quality check**	
Sequencing depth/read length	50Gbp/101bp pair end
Total read-pairs	304 million
Percentage achieving a quality score of Q30 (equivalent to 1 mistake in 1,000 bps)	72%
Mapping rate (to mouse reference mm10)	93.0%
Percentage of valid barcodes	97.7%
Sequencing saturation	77.2%
Fraction of reads in cells and beads	100%
Cells	*90.9%*
Background beads	*9.1%*
**Transcriptome mapping**	
Total number of genes detected in aggregate	18,247
Total number of UMIs detected	46,239,500
Number of reads (R2) per UMI detected	6.6 (=304million/46million)
Total number of cells captured and sequenced	5,649
Average number of gene detected	2,058 (s.d. 637 genes)
Number of UMIs per cell	6,601
Blood cell captured	189
Average number of genes detected in blood cell	216
Non-blood cells	5,460 (=5649-189)
Total number of Lgr5+ cell detected	207
Express normal Lgr5 allele only	*94*
Express Lgr5-CRE-ERT2 allele only	*87*
Express both alleles	*26*
**Data analysis**	
DEG stringency	• FDR cutoff of <0.05.<br>• expression difference at least 20% (defined as %{turned on in cluster_i} - %{turned on in all_other_clusters})
Clustering of dispersed genes (Heatmap)	• Top 5% dispersed gene (genome-wide)<br>• at least 50 cells expressed
Pseudotime trajectory	• Negative binomial distribution<br>• based on the genome-wide UMI count data for genes expressing in 10 cells or more

Table S6. DEGs "Cluster 1 vs non-cluster 1" and "Lgr5+ vs Lgr5-" (Single cell transcriptome of E14.5 knee joint). Related to Figure 4

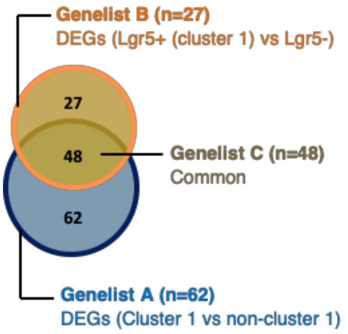

**Genelist A (n=62)**	***Osr2***, *Man1a, Nbl1, Angptl2, Dusp1, Egfl6, Gas2, Rbms3,* ***Creb5***, *Rnd3,* ***Zfp503***, *Dclk1, F2r,* ***Zfp36l1***, *Igfbp4, 3632451o06rik, Epb41l3, Svil, Tpm1, Palld, Tuba1a, Hmcn1, Cdh11, Hspa1a, Pdgfra, Cpq, Epha7,* ***Foxp1***, *Loxl2, Csrp1, Dbn1, Col6a1, Col8a2, Vcan, Ddah2, Col6a2, Cdon,* ***Prrx1***, *Tagln2, Sulf2, Col5a2,* ***Tax1bp3***, *Zyx, Nrep, Ltbp3,* ***Nfia***, *Rcan1, Fbln1, Igfbp7, Arhgap31, Marcksl1, Kctd12,* ***Hoxa10***, *Abracl, Marcks, Hmgb3, Timp2, Map1lc3a, Ifitm3, Aes,* ***Trps1, Barx1***
**Genelist B (n=27)**	*F2rl1, Fhod3, Adam33, Tagln, Fst, Khdrbs2, Hspb8, Chst5, Inhba, Tnni2, Etl4,* ***Meox2***, *Sorcs2, Tlr2, Bmp4, Gfra4, Syt11,* ***Mafb***, *Ppm1l, Pdgfrl, Gpm6b, Fjx1, Tmem119, Pcdh18, Enpp1, Rgcc, Pdgfc*
**Genelist C (n=48)**	*Lrrc17, Pamr1, Htra1, Cdh13, Phactr1, Aqp1, Tm4sf1, Nupr1, Tcea3, Lrrn1, Sfrp2, Ccdc80, Cntfr, Tgfbi, Ssc5d, Gas1*, ***Pax9***, ***Mkx***, *Pid1, Map1b, Gdf5, Dkk3, Sulf1, Gm26771, Dusp8, Mylk, Shisa2, Adamtsl1, Prss23, Krt17, Cited2, Wisp1, Loxl1,* ***Sox4,*** *Lmo1, Ackr3,* ***Bcl11a***, ***Scx***, *Phldb2, Myo1b, Spint2, Fbn2, Postn, Mfap4, Col12a1, Tgfb2, Fn1, Ier3*

Bold = transcription factors

Table S7. Primers used in this paper

	Forward primer	Reverse primer
**Primers for genotyping Lgr5-eGFP-IRES-CreER^{T2} mice**		
wildtype	5'-CTGCTCTCTGCTCCCAGTCT-3'	5'-ATACCCCATCCCTTTTGAGC-3'
mutant		5'-GAACTTCAGGGTCAGCTTGC-3'
**Primers for genotyping R26R-lacZ mice**		
wildtype	5'-AAAGTCGCTCTGAGTTGTTAT-3'	5'-GGAGCGGGAGAAATGGATATG-3'
mutant		5'-GCGAAGAGTTTGTCCTCAACC-3'
**Primers for Quantitative PCR**		
Lgr4	5'-CACAACCTGTCTGATCTGCAT-3'	5'-TGGTGCTTATTTTTGTCCCTGT-3'
Lgr5	5'-CACAGCCACTGCGGCGACTT-3'	5'-CAATGGGCGTCTGCCGGGTC-3'
Lgr6	5'-CTCCTTGCTGGACAAGCTGA-3'	5'-TCTCAAAGAGGTGCTCGCAG-3'
Gdf5	5'-AAAGGGAGGTAACAGCAGCG-3'	5'-CCCATCCTTCTCCAAGGCAC-3'
Cd90	5'-TCTCCTGCTCTCAGTCTTGC-3'	5'-TATTCTCATGGCGGCAGTCC-3'
Cd105	5'-TAGCACCTTGTCCCAGGAAG-3'	5'-CAGTACAGAGGGCAGGACAA-3'
Notch1	5'-TGTGAGTCCAACCCTTGTGT-3'	5'-CGTTGATGTTGGTCTGGCAA-3'
Klf4	5'-AAAAGAACAGCCACCCACACTT-3'	5'-CCCAGTCACAGTGGTAAGGTTTC-3'
Gapdh	5'-TGCACCACCAACTGCTTAG-3'	5'-GATGCAGGGATGATGTTC-3'
